# Health insurance participation may mitigate the health impact of food insecurity among Chinese working-age adults

**DOI:** 10.3389/fpubh.2026.1808940

**Published:** 2026-04-20

**Authors:** Kaiyin Xu, Fei Men, Yuxuan Fan, Guofan He

**Affiliations:** Key Laboratory of Environmental Medicine Engineering, Ministry of Education, Department of Health Insurance, School of Public Health, Southeast University, Nanjing, China

**Keywords:** food insecurity, health inequity, health insurance, healthcare access, working-age adults

## Abstract

In mainland China, approximately 250 million people are unable to afford healthy meals. Food insecurity has been linked to poorer health outcomes in western countries, yet its impact remains understudied in China, especially among working-age adults. We analyzed cross-sectional data from an online survey conducted in 2024 with 4,795 individuals aged 30–60 from three Chinese provinces. Respondents self-reported chronic conditions, mental disorders, disabilities, and doctor visits. Household food insecurity was assessed by the FAO’s Food Insecurity Experience Scale (FIES). The results showed that food insecurity was positively associated with chronic conditions, mental disorders, disabilities, and doctor visits in a dose–response fashion after confounders adjustment. Health insurance participation mitigated the association of food insecurity with chronic conditions and doctor visits. Healthcare accessibility, dietary quality, social support, and psychological chronic stress partially mediated the food insecurity-health relationship. Efforts to broaden and upgrade health insurance coverage and promote healthcare accessibility may help reduce health inequity across food security levels.

## 1 Introduction

Household food insecurity, the limited or uncertain ability to acquire nutritious and safe foods in socially acceptable ways (1), affects approximately 733 million people globally (2). In mainland China, 17.5% of the population, or approximately 250 million people, were unable to afford healthy meals in 2022 (2).

Interests have been growing in the role of food insecurity as a potential social determinant of health (3, 4). Evidence has linked food insecurity to various adverse health conditions in the US and Canada (5), including chronic diseases (6), mental disorders (7), and disabilities (8–10). A dose–response relationship was observed, with severer food insecurity associated with worse health outcomes (11, 12). One explanation for the food insecurity-health relationship is the “nutrition quality gap” (13), whereby food-insecure families often have nutritionally inadequate diets and poorer dietary quality due to tight budgets, leading to other health issues (14, 15). Another possibility is that food-insecure families often experience chronic stress and face the difficult trade-off between food purchase and health management (16, 17), which could in turn jeopardize food-insecure adults’ health (18–21). In addition, limited social support may further exacerbate the health consequences of food insecurity by reducing individuals’ ability to cope with economic strain and maintain healthy behaviors (22). Health insurance may mitigate the impact of food insecurity on health by fostering access to medical treatment and health management while keeping the out-of-pocket medical costs to a reasonable level (23). Expanded health insurance has been associated with decreased food insecurity (24) and better chronic disease management in the US (25).

Empirical research on household food insecurity is scant in China: the few exceptions focused on the older adults and children, linking food insecurity to multimorbidity and depression among older adults (26, 27) and anemia and malnutrition among children (28, 29). Moreover, the past studies have mostly relied on the short form food insufficiency questionnaire (26, 27, 29), making it impossible to examine the graded effect of food insecurity by severity levels. The mechanisms by which food insecurity might impact health remains understudied, especially with regard to the role of health insurance and healthcare accessibility as well as various psychosocial and behavioral pathways. This study fills the gaps by analyzing data from a recent online survey administered to working-age adults in China. To our knowledge, this is the first study to assess the prevalence and severity of food insecurity of working-age adults in China. The findings could advance our understanding of health disparities and potential remedies thereof.

## 2 Methods

### 2.1 Study sample

We administered a cross-sectional survey on Wenjuanxing—the leading online survey platform in China—from June to September, 2024 (Supplementary file 1). Wenjuanxing’s sampling service distributed our survey to registered participants from their nationwide respondent pool. We limited our sample to those aged 30–60 with a personal monthly income up to ¥10,000 RMB living in Jiangsu, Shandong or Sichuan. The three provinces combined account for roughly 1/5 of China’s population, and they represented the lower-than-average, average, and higher-than-average levels of per capita GDP at the provincial level, respectively. The survey used a structured questionnaire to collect data on household food security, individual health, and other sociodemographic information. We employed reverse coding, time stamps, among other measures of quality control for the survey and oversampled those without a college degree to better capture the otherwise hard-to-reach disadvantaged population. The raw data contained 4,845 observations. We excluded 50 individuals aged below 30 or over 60, and obtained a sample with 4,795 working-age adults.

### 2.2 Variables

#### 2.2.1 Outcome measures

The key outcomes concern four aspects of a respondent’s health, namely chronic conditions, mental disorders, disabilities, and doctor visits for illnesses and injuries. Respondents were asked about prior diagnosis of chronic conditions (Hypertension, Hyperlipidemia, Hyperglycemia, Diabetes, Malnutrition, Anemia, Hyperuricemia, Osteoporosis, Intervertebral Disc Diseases, Cancer, Cerebrovascular Diseases, Heart Diseases, Respiratory Diseases, Gastrointestinal Diseases, Urinary System Diseases, Endocrine System Diseases, Rheumatic Diseases, Neurological Disorders) and mental disorders (Major Depression, General Anxiety Disorder, Bipolar Disorder, Insomnia, Obsessive-Compulsive Disorder, Schizophrenia, Personality Disorder, Anorexia). They were also inquired about different types of disabilities (physical, mental, visual, speech-hearing, or intellectual disabilities including autism spectrum disorder) and reasons for doctor visits in the past year (Chronic Conditions, Acute Conditions, Mental Disorders, Unintentional Injuries, Intentional Injuries). Answers to those questions were dichotomized. We also counted the number of reported chronic conditions, mental disorders, disabilities, and reasons for recent doctor visits, respectively, to examine multimorbidity and other health complications.

#### 2.2.2 Food insecurity measure

The core explanatory variable was household food insecurity, measured by the FAO’s Food Insecurity Experience Scale (FIES) (30). FIES captures households’ lived experiences of food insecurity through eight questions about compromised food quality and quantity due to resource constraints (Supplementary Table A). Affirmative answers to each question add one point to the scale. We coded FIES scores into four levels: 0 (food security), 1–3 (mild food insecurity), 4–6 (moderate food insecurity), and 7–8 (severe food insecurity). We also tested alternative classifications of the variable including a three-level version (0 = secure, 1–3 = mild, 4–8 = moderate–severe), and two binary versions (strict: 0 vs. 1–8; lenient: 0–1 vs. 2–8) for sensitivity checks.

The reliability and validity of the Food Insecurity Experience Scale were assessed in the study sample. The scale demonstrated good internal consistency (Cronbach’s *α* = 0.86). Confirmatory factor analysis supported the unidimensional structure of the scale, with acceptable model fit indices (CFI = 0.98, TLI = 0.97, RMSEA = 0.07, SRMR = 0.03). All standardized factor loadings exceeded 0.50 (Supplementary Table B).

#### 2.2.3 Social health insurance participation measure

The social health insurance system in China comprises two schemes, jointly covering 95% of population: the Urban Employee Basic Medical Insurance (UEBMI), covering 371 million urban workers, and the Urban and Rural Resident Basic Medical Insurance (URRBMI), covering 963 million agricultural workers and the unemployed (31). UEBMI is funded by employees and their employers through wage deduction, with little government subsidy, while URRBMI is mainly subsidized by the government, with individuals contributing a flat annual fee on top. The contribution, service coverage and cost reimbursement are all greater in UEBMI versus URRBMI (32) (Supplemntary eTable C).

We coded the social health insurance participation as a categorical variable, distinguishing the UEBMI participants from the URRBMI participants, participants in other insurance schemes (such as private insurance), and the uninsured.

#### 2.2.4 Control variables

In line with prior literature (5, 6, 33), we adjusted for a number of confounding factors in our models, including respondents’ sociodemographic characteristics (sex, age, residence, ethnicity, province, education, marital status, number of children, number of adults), economic conditions (family income, liquid assets, fixed assets, debt), and health-related lifestyle (smoking status, drinking frequency). All covariates were coded as binary or categorical variables.

We also created several binary variables for health care delayed or forgone due to cost, diminished food expenditure, lower subjective cognition of dietary health, social support deficit (assessed by timely support unavailability, loneliness, and family functional impairment due to poor health/disability), and psychological chronic stress overload (defined by at least one of depressive symptoms, anxiety, uncontrollable worry, or poor sleep quality), respectively, to test their mediating effects in the food insecurity-health relationship.

### 2.3 Statistical analyses

**Figure 1:**
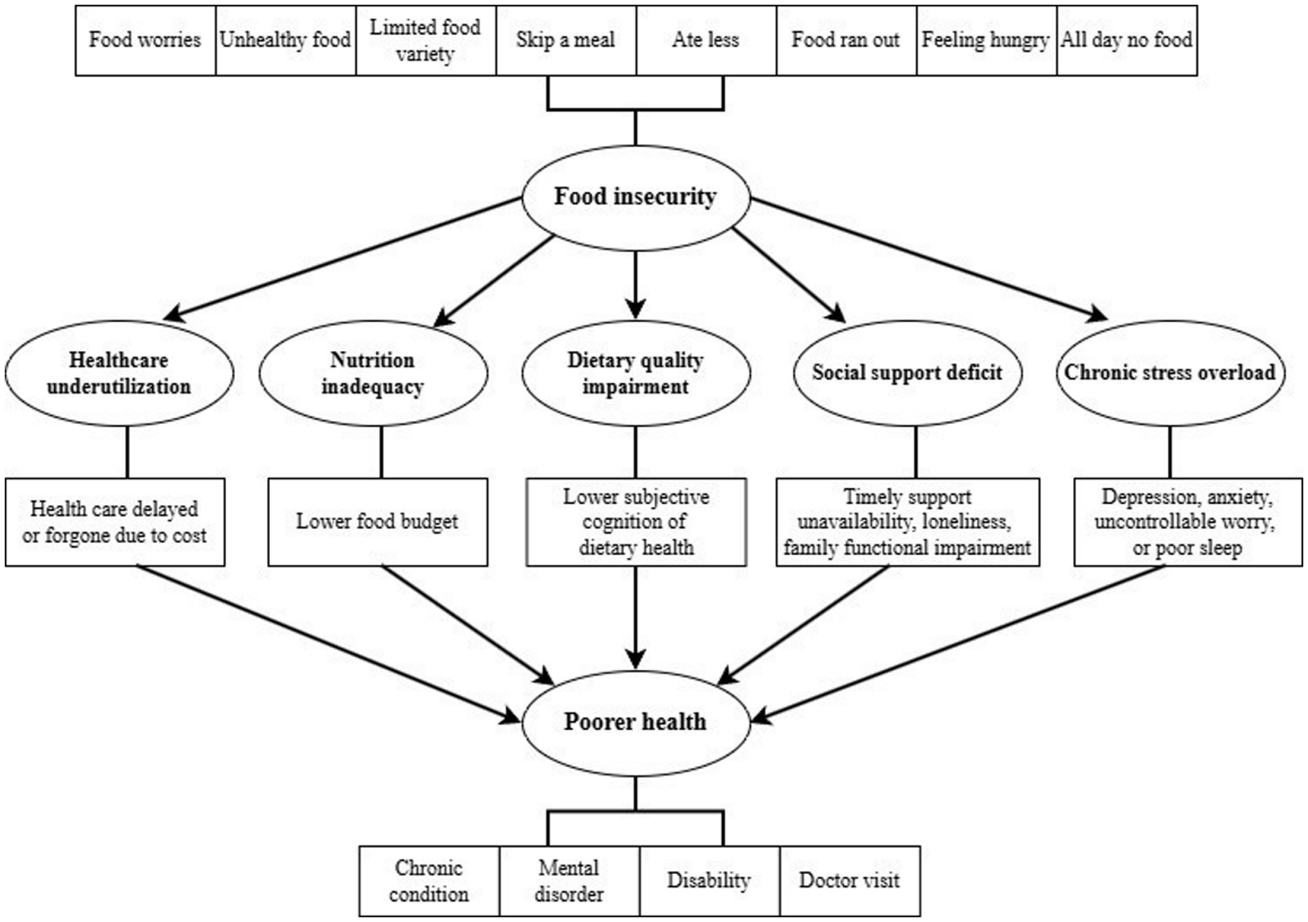
The GSEM model including the potential mediators in the food insecurity-health relationship.

We employed logistic regressions and Poisson regressions to estimate the association between food insecurity and health outcomes, including the presence and number of different chronic conditions, mental disorders, disabilities, and doctor visits. We also estimated the interaction effects between food insecurity and health insurance participation, converting significant interaction terms to predicted probabilities and marginal effects for easier interpretation. Generalized Structural Equation Modeling (GSEM) was further employed to test the potential mediating effect of healthcare accessibility, food budget, dietary quality, social support, and psychological chronic stress on the food insecurity-health relationship (Figure 1).

Missing values were few and we kept them in the models as separate categories of the covariates. Removing individuals with missing values in covariates had little impact on the findings (Supplementary Table D). To assess the robustness of our findings to potential selection bias and unmeasured confounding, we conducted bounds analysis following Oster’s approach (34). We calculated coefficient stability under varying assumptions about selection on unobservables relative to selection on observables and maximum R-squared values. This analysis provides bounds for the true causal effect under different assumptions about unmeasured confounding factors (Supplementary Table E). To address the potential bias arising from the online non-probability sampling, post-stratification sampling weights were calculated based on the population distributions of age group, sex, residence, and province using data from the China’s Seventh National Population Census. The weights were constructed as the ratio of population proportions to sample proportions within each stratum.

All analyses used Stata SE 18.0 with two-sided confidence intervals and *p* < 0.05 significance threshold (35). The study was approved by the Independent Ethics Committee for Clinical Research of Zhongda Hospital Affiliated to Southeast University under the protocol #2024ZDSYLL292-P01.

## 3 Study results

### 3.1 Sample characteristics

Food-insecure individuals showed significantly higher rates of adverse health outcomes compared to food-secure individuals, with notably elevated prevalence of chronic diseases, mental illness, disabilities, and doctor visits (Supplementary Table F).

Food-insecure individuals differed significantly from their food-secure counterparts in sociodemographic and economic characteristics as well as health-related lifestyle (Table 1). Relative to their food-secure peers, food-insecure individuals were more likely to come from rural areas, have no college degree, be unmarried, live with more children and adults, lack health insurance, have lower income and assets, carry more debt, smoke, and drink alcohol regularly.

**Table 1:** Sociodemographic characteristics, economic status, and lifestyles, overall and by food insecurity status.

Variables		No. (%)			*p*
		Total (*n* = 4,795)	Food secure (*n* = 3,399)	Food insecure (*n* = 1,396)	
Age	31–35	2,678 (55.8)	1869 (55.0)	809 (58.0)	0.030
	36–40	1,196 (24.9)	845 (24.9)	351 (25.1)	
	41–60	921 (19.2)	685 (20.2)	236 (16.9)	
Sex	Female	2,660 (55.5)	1898 (55.8)	762 (54.6)	0.427
	Male	2,135 (44.5)	1,501 (44.2)	634 (45.4)	
Residence	Rural	1,380 (28.8)	837 (24.6)	543 (38.9)	<0.001
	Urban	3,415 (71.2)	2,562 (75.4)	853 (61.1)	
Ethnicity	Han	4,725 (98.5)	3,351 (98.6)	1,374 (98.4)	0.668
	Minority	70 (1.5)	48 (1.4)	22 (1.6)	
Province	Jiangsu	1722 (35.9)	1,262 (37.1)	460 (33.0)	0.004
	Shandong	1741 (36.3)	1,187 (34.9)	554 (39.7)	
	Sichuan	1,332 (27.8)	950 (27.9)	382 (27.4)	
Education	High school incomplete	142 (3.0)	65 (1.9)	77 (5.5)	<0.001
	High school diploma	749 (15.6)	469 (13.8)	280 (20.1)	
	College degree	3,436 (71.7)	2,509 (73.8)	927 (66.4)	
	Graduate degree	468 (9.8)	356 (10.5)	112 (8.0)	
Marital status	Unmarried	622 (13.0)	395 (11.6)	227 (16.3)	<0.001
	Married	4,006 (83.5)	2,902 (85.4)	1,104 (79.1)	
	Divorced/separated/widowed	167 (3.5)	102 (3.0)	65 (4.7)	
Number of children	No children	858 (17.9)	606 (17.8)	252 (18.1)	<0.001
	1 child	2,863 (59.7)	2071 (60.9)	792 (56.7)	
	2 children	1,006 (21.0)	692 (20.4)	314 (22.5)	
	3 or more children	68 (1.4)	30 (0.9)	38 (2.7)	
Number of adults	1 adult	283 (5.9)	181 (5.3)	102 (7.3)	<0.001
	2 adults	2,470 (51.5)	1812 (53.3)	658 (47.1)	
	3 adults	1,005 (21.0)	677 (19.9)	328 (23.5)	
	4 or more adults	1,037 (21.6)	729 (21.4)	308 (22.1)	
Health insurance	Uninsured	89 (1.9)	40 (1.2)	49 (3.5)	<0.001
	UEBMI	3,581 (74.7)	2,719 (80.0)	862 (61.7)	
	URRBMI	956 (19.9)	536 (15.8)	420 (30.1)	
	Only other types of health insurance	169 (3.5)	104 (3.1)	65 (4.7)	
Family income	¥0–4 k	249 (5.2)	88 (2.6)	161 (11.5)	<0.001
	¥4–9 k	583 (12.2)	285 (8.4)	298 (21.3)	
	¥9–15 k	1,451 (30.3)	1,003 (29.5)	448 (32.1)	
	Above ¥15 k	2,440 (50.9)	1974 (58.1)	466 (33.4)	
	Unknown	72 (1.5)	49 (1.4)	23 (1.6)	
Fixed assets	¥0–40 k	351 (7.3)	141 (4.1)	210 (15.0)	<0.001
	¥40–200 k	634 (13.2)	342 (10.1)	292 (20.9)	
	¥200–1,000 k	1,477 (30.8)	1,003 (29.5)	474 (34.0)	
	Above ¥1,000 k	2078 (43.3)	1737 (51.1)	341 (24.4)	
	Unknown	255 (5.3)	176 (5.2)	79 (5.7)	
Liquid assets	Below ¥5 k	321 (6.7)	129 (3.8)	192 (13.8)	<0.001
	¥5–20 k	590 (12.3)	310 (9.1)	280 (20.1)	
	¥20–100 k	969 (20.2)	604 (17.8)	365 (26.1)	
	Above ¥100 k	2,608 (54.4)	2,139 (62.9)	469 (33.6)	
	Unknown	307 (6.4)	217 (6.4)	90 (6.4)	
Debts	No debt	2,203 (45.9)	1772 (52.1)	431 (30.9)	<0.001
	¥1–10 k	277 (5.8)	128 (3.8)	149 (10.7)	
	¥10–100 k	563 (11.7)	319 (9.4)	244 (17.50)	
	Above ¥100 k	1,617 (33.7)	1,086 (32.0)	531 (38.0)	
	Unknown	135 (2.8)	94 (2.8)	41 (2.9)	
Smoking status	Never smoked	3,342 (69.7)	2,424 (71.3)	918 (65.8)	<0.001
	Quit smoking	709 (14.8)	438 (12.9)	271 (19.4)	
	Currently smoking	744 (15.5)	537 (15.8)	207 (14.8)	
Drinking frequency	Do not drink	1,548 (32.3)	1,149 (33.8)	399 (28.6)	0.002
	Less than once a month	1,215 (25.3)	857 (25.2)	358 (25.6)	
	Once or twice a month	1,133 (23.6)	768 (22.6)	365 (26.1)	
	More than once a week	899 (18.7)	625 (18.4)	274 (19.6)	

*p* is the *p*-value for the chi-square tests for the difference in summary statistics between the food-secure and food-insecure individuals.

### 3.2 Association between food insecurity and aggregated health outcomes

Table 2 presented the results of fully adjusted logistic regressions (complete output in Supplementary Table G) and Poisson regressions. Compared to food-secure individuals, those with mild, moderate, and severe food insecurity were 2 (adjusted odds ratio [AOR] = 2.01; CI, 1.50–2.69), 3.8 (AOR = 3.82; CI, 2.29–6.38), and 2.2 (AOR = 2.15; CI, 1.10–4.18) times more likely to be diagnosed with chronic conditions, respectively. The mild, moderate, and severe food insecurity were linked to 2.2 (AOR = 2.22; CI, 1.53–3.21), 4.8 (AOR = 4.80; CI, 2.94–7.85), and 6.9 (AOR = 6.87; CI, 3.65–12.93) times higher odds of diagnosis of mental disorders relative to food security, respectively. And the mild, moderate, and severe food insecurity were associated with 1.9 (AOR = 1.88; CI, 1.17–3.02), 4.4 (AOR = 4.42; CI, 2.52–7.78), and 7.4 (AOR = 7.39; CI, 3.48–15.67) times higher odds of carrying disabilities in the past year, respectively. Moreover, the mildly, moderately, and severely food-insecure adults were 1.7 (AOR = 1.72; CI, 1.29–2.27), 2.3 (AOR = 2.33; CI, 1.45–3.74), and 3.2 (AOR = 3.22; CI, 1.63–6.36) times more likely to visit doctors in the past year, respectively.

**Table 2:** The correlation between 4-category household food insecurity and the presence and number of health outcomes.

Variable	The presence of health outcomes (*n* = 4,795) AOR [95% CI]				The number of different health outcomes (*n* = 4,795) IRR [95% CI]			
	Any chronic condition	Any mental disorder	Any disability	Any doctor visit	Number of chronic conditions	Number of mental disorders	Number of disabilities	Number of reasons for doctor visits
Food security	1.00	1.00	1.00	1.00	1.00	1.00	1.00	1.00
	[Reference]	[Reference]	[Reference]	[Reference]	[Reference]	[Reference]	[Reference]	[Reference]
Mild food insecurity	2.01***	2.22***	1.88**	1.72***	1.41***	2.09***	1.81**	1.38***
	[1.50, 2.69]	[1.53, 3.21]	[1.17, 3.02]	[1.29, 2.27]	[1.19, 1.66]	[1.53, 2.85]	[1.20, 2.74]	[1.17, 1.63]
Moderate food insecurity	3.82***	4.80***	4.42***	2.33***	2.00***	3.21***	3.44***	2.03***
	[2.29, 6.38]	[2.94, 7.85]	[2.52, 7.78]	[1.45, 3.74]	[1.61, 2.50]	[2.29, 4.52]	[2.16, 5.49]	[1.59, 2.58]
Severe food insecurity	2.15*	6.87***	7.39***	3.22***	2.59***	3.71***	5.61***	2.45***
	[1.10, 4.18]	[3.65, 12.93]	[3.48, 15.67]	[1.63, 6.36]	[1.83, 3.66]	[2.56, 5.37]	[3.51, 8.97]	[1.79, 3.35]

AOR, adjusted odds ratios; IRR, incidence rate ratio; CI, 95% confidence intervals in brackets. All models were weighted with post-stratification sampling weights and adjusted for age, sex, residence, ethnicity, province, education, number of children, marital status, number of adults, health insurance participation, family income, fixed assets, liquid assets, debt, smoking status, and drinking frequency. ^*^p < 0.05, ^**^p < 0.01, ^***^p < 0.001.

The dose–response relationship was also found with counted number of chronic conditions, with mild, moderate, and severe food insecurity linked to 1.4 (incidence rate ratio [IRR] = 1.41; CI, 1.19–1.66), 2 (IRR = 2.00; CI, 1.61–2.50), and 2.6 (IRR = 2.59; CI, 1.83–3.66) times higher count of chronic conditions relative to food security, respectively. Compared to food-secure individuals, those with mild, moderate, and severe food insecurity were also more likely to report higher counts of mental disorders, disabilities, and different reasons for doctor visits.

We conducted several robustness checks. We first tested alternative classifications of food security status: a three-tiered scale and two variations of binary classifications. Given the potential overdispersion and zero inflation issues with the count outcome variables, we also tested the negative binomial regression model and the zero-inflated Poisson model. All results were qualitatively identical to our main findings (Supplementary Tables H–L). We also tried excluding family income and liquid assets from the model given their potential mediating effect on the food insecurity-health relationship; results largely stayed unchanged (Supplementary Table M).

### 3.3 Associations between binary food insecurity status and disaggregated health outcomes

**Figure 2:**
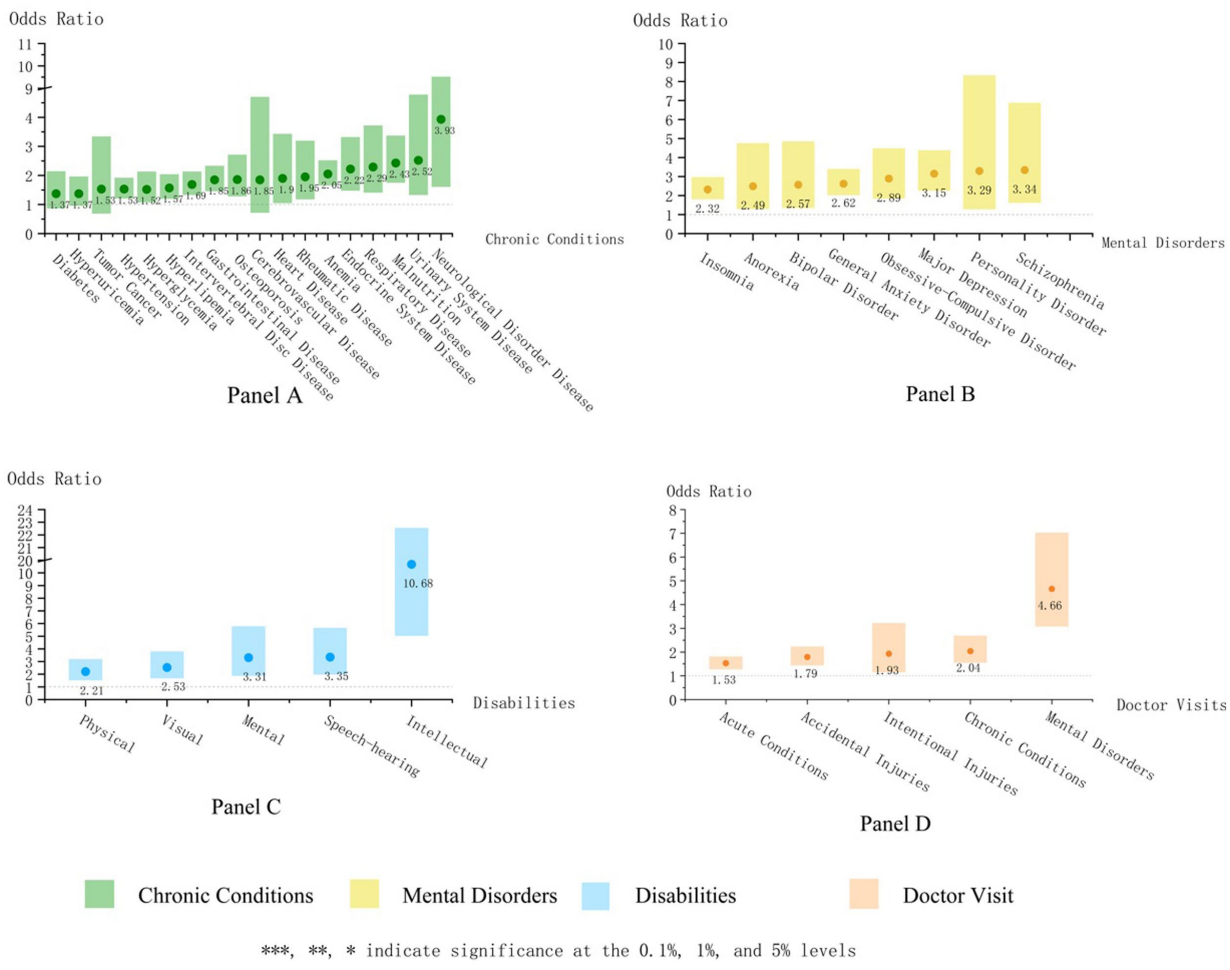
Forest plot of the adjusted odds ratios of food insecurity in models predicting health.

Food-insecure individuals showed significantly higher odds of reporting most chronic conditions under examination compared to their food-secure counterparts (Figure 2; Supplementary Table N). Notable associations were observed in neurological disorders (AOR = 3.93; CI, 1.62–9.50), urinary diseases (AOR = 2.52; CI, 1.34–4.77), and malnutrition (AOR = 2.43; CI, 1.76–3.36). Diabetes, gout, cancers, and cerebrovascular diseases showed positive yet non-significant associations with food insecurity.

Food-insecure individuals demonstrated significantly higher odds of all measured mental health conditions compared to food-secure individuals (Supplementary Table O), including Major Depression (AOR = 3.15; CI, 2.27–4.36), General Anxiety Disorder (AOR = 2.62; CI, 2.04–3.38), and insomnia (AOR = 2.32; CI, 1.82–2.95).

Food-insecure individuals also showed significantly higher odds of carrying all types of disabilities compared to food-secure ones (Supplementary Table P), including mental (AOR = 3.31; CI, 1.90–5.75), speech-hearing (AOR = 3.35; CI, 2.00–5.63), visual (AOR = 2.53; CI, 1.70–3.77), and physical disabilities (AOR = 2.21; CI, 1.55–3.16). Intellectual disability (AOR = 10.68; CI, 5.06–22.52) showed a particularly strong link with food insecurity, though the results were based on few observations.

Relative to their food-secure counterparts, food-insecure individuals were more likely to visit doctors in the past year regardless of reasons (Supplementary Table Q). The strongest association was found between food insecurity and visits due to mental disorders (AOR = 4.66; CI, 3.09–7.02), while visits driven by other diseases and injuries showed less pronounced yet significant association with food insecurity.

### 3.4 Moderating effects of health insurance participation

Participation in UEBMI and URRBMI significantly mitigated the association of any food insecurity with having any chronic condition (interaction IRR = 0.21–0.26, *p* < 0.05), having more chronic conditions (interaction IRR = 0.23–0.28, *p* < 0.05), and visiting doctors due to more reasons (interaction IRR = 0.43–0.45, *p* < 0.05) (Supplementary Tables R, S).

To provide more intuitive interpretation of our findings, we presented the average marginal effects of binary food insecurity status across different insurance categories (Table 3). Food insecurity was associated with 47% higher likelihood of having chronic conditions (AME = 0.47, *p* < 0.001), 1.5 fewer chronic conditions (AME = 1.50, *p* < 0.001) and 0.43 fewer reasons for doctor visits (AME = 0.43, *p* < 0.01) among the uninsured individuals. The comparable changes in risks and numbers were much lower for the participants of UEBMI (AME = 0.15, 0.37 and 0.21, *p* < 0.001) and URRBMI (AME = 0.20, 0.47and 0.24, *p* < 0.001). Food-insecure individuals had lower predicted probabilities of having chronic conditions and fewer expected chronic conditions when insured versus uninsured, while food-secure individuals had more expected doctor visits when insured versus uninsured (Supplementary Table T).

**Table 3:** Average marginal effects (AME) of food insecurity on chronic conditions and the number of reasons for doctor visits.

Variable	Any chronic condition	Number of chronic conditions	Number of reasons for doctor visits
(*n* = 4,795)			
AME of food insecurity			
Uninsured	0.47***	1.50***	0.43**
	(0.09)	(0.43)	(0.14)
UEBMI	0.15***	0.37***	0.21***
	(0.02)	(0.05)	(0.03)
URRBMI	0.20***	0.47***	0.24***
	(0.03)	(0.09)	(0.05)
Only other types of health insurance	0.15	0.29	0.29**
	(0.08)	(0.19)	(0.10)

Standard errors in parentheses.

All models were weighted with post-stratification sampling weights and adjusted for age, sex, residence, ethnicity, province, education, number of children, marital status, number of adults, family income, fixed assets, liquid assets, debt, smoking status, and drinking frequency. The marginal effects were calculated based on the Model 1 from Supplementary Table R and the Models 1, 4 from Supplementary Table S. ^*^p < 0.05, ^**^p < 0.01, ^***^p < 0.001.

### 3.5 Mediating effect of healthcare accessibility

We estimated the mediating effects of cost-related healthcare underuse, food expenditure, lower subjective cognition of dietary health, social support deficit, and chronic stress overload with the GSEM model. Delaying or forgoing health care due to cost mediated the correlations between food insecurity and all four health outcomes, with especially strong effect for disabilities (AOR = 2.27) and mental disorders (AOR = 2.12). The association of diminished food expenditure with health outcomes was trivial (*p* > 0.05). However, Lower subjective cognition of dietary health significantly mediated the associations with chronic conditions (AOR = 0.47), mental disorders (AOR = 0.64), and doctor visits (AOR = 0.67). Social support deficit contributed to elevated risks of chronic conditions (AOR = 1.61), mental disorders (AOR = 3.12), and disabilities (AOR = 2.35). Psychological chronic stress overload emerged as the strongest mediator across all outcomes, driving particularly robust associations with mental disorders (AOR = 6.10), and chronic conditions (AOR = 3.10) (Supplementary Table U). The AIC and BIC were similar across the models (Supplementary Table V).

## 4 Discussion

### 4.1 Results discussion

This study examined the relationship between food insecurity and health among working-age adults in China using online survey data from three major provinces. We found strong graded associations between food insecurity and various adverse health outcomes, with health insurance moderating the relationship and healthcare accessibility mediating the same.

Food-insecure individuals showed significantly higher risks of various chronic diseases and mental illnesses, consistent with previous research (6, 8). We extended existing literature to the context of an emerging economy, identifying specific conditions vulnerable to food insecurity, including cardiovascular, respiratory, and mental disorders, thus adding evidence to the hypothesis on the comprehensive health impact of food insecurity. However, associations for diabetes, hyperuricemia, cancer, and cerebrovascular diseases were non-significant. This likely stem from limited statistical power due to low prevalence, or potential dietary confounding—for instance, lower purine intake among food-insecure individuals may mask risks for gout. Additionally, the long latency of malignancy and stroke might result in lower sensitivity within our working-age cohort. Finally, the inherent selection bias of online surveys may have excluded severely ill patients with limited digital access, leading to a potential underestimation of these associations. Notably, these associations persisted largely independent of income and liquid assets, suggesting food insecurity may influence health through multiple pathways beyond sheer economic constraints (36, 37). Healthcare accessibility, partly determined by health insurance participation, was one such mediator we identified, which also aligned with prior literature (18, 19, 38). Chronic stress also emerged as an important mediator, supporting previous studies showing that persistent stress related to financial strain and food uncertainty may deteriorate health (17). Social support showed a modest mediating effect, suggesting that stronger social networks may help buffer the negative health consequences of food insecurity (22). Dietary quality also partially mediated the food insecurity-health relationship, and the fact that food expenditure did not mediate the food insecurity-health relationship suggests that food insecurity was a more nuanced issue than nutritional deficiency or reduced food spending (16). In the context of China’s economic transition, food-insecure households may cope with financial constraints by choosing cheaper, energy-dense but nutrient-poor foods, such as highly processed products, rather than reducing overall food expenditure. Such dietary patterns can still compromise nutritional quality and contribute to adverse health outcomes.

Food insecurity was strongly associated with higher likelihood of having various disabilities and doctor visits due to all types of illnesses and injuries aligns with previous findings linking severe food insecurity to disabilities and hospital admissions in developed countries (6, 12). Those with mental disorders may be especially disadvantaged when it comes to food security reduction. Similar to the study in Canada (6), mental disorders emerged as one of the strongest health correlates of food insecurity.

While our findings suggested that food insecurity was associated with poorer health, longitudinal evidence from other contexts indicated a bidirectional cycle. Food insecurity has been shown to be a potent predictor of the subsequent onset of physical and mental conditions (39), as well as higher healthcare use and costs in succeeding years (7). Conversely, health issues posed further barriers to food security enhancement through imposing additional living costs and reducing labor market participation or earning capacity (5). This bidirectional link warrants prioritized investigation in the Chinese working-age population, who may be particularly vulnerable to falling into a trap where nutritional deprivation and physical decline mutually exacerbate one another over time.

The fact that health insurance significantly moderated the relationship between food insecurity and chronic conditions may be attributed to reduced financial barriers to enhanced healthcare access (40). Food-insecure individuals often face difficult trade-offs between food and medical expenses (23), and health insurance participation could make management of chronic conditions more affordable (25). That health insurance mitigated the association between food insecurity and doctor visits may be driven by improved self-care and reduced need for emergency room visits (21). As the public health strategy moves its focus from treatment to prevention in China and elsewhere (41), we can expect health insurance to play a bigger role in further reducing usage of high-cost healthcare services through stronger preventive initiatives.

We found no moderating effect of health insurance on mental disorders or disabilities. Cultural stigma, treatment complexity, and high recurrence rates create important barriers for accessing mental healthcare (42–44). Even insured families often face significant economic burdens from mental disorders treatment. In China, approximately 130 million adults suffer from mental disorders (45), and the cost of mental illnesses accounts for more than 15% of the national healthcare expenditure and 1.1% of the GDP (46). Similarly, about half of China’s disabled population reported unmet rehabilitation needs and about one-fifth needed more healthcare (47). Improving the current health insurance schemes is key to reduce the economic burden of people with disabilities and enhancing their access to healthcare service (48).

The interplay between food insecurity and health in China reflects broader challenges facing middle-income countries undergoing rapid socioeconomic transitions. Fortunately, food insecurity is modifiable through policy interventions. Clinicians may incorporate the food insecurity questions into patient interviews to identify individuals at risk of food insecurity (49). The government may consider expanding social health insurance coverage and increasing the insurance benefit level -such as reducing copay for low-income patients - to better address the healthcare needs of food-insecure individuals (50). Moreover, the government may assess whether the social assistance programs targeting the economically disadvantaged families have adequate coverage and benefits; the current policies have largely overlooked the working poor despite their increasing prevalence (51). Integrated policy interventions that treat food security as a part of the solutions to public health challenges hold promise for fostering population health, reducing health inequities, and improving the sustainability of the healthcare system.

### 4.2 Limitations

This study has several limitations. First, the cross-sectional design precluded causal inference or the observation of long-term dynamic changes in food security status. While we controlled for a wide range of socio-economic confounders, the potential for reverse causality cannot be entirely ruled out. Future research should prioritize the use of longitudinal panel data to better disentangle the temporal precedence and causal pathways between food insecurity and health. For the Chinese working-age population, targeted longitudinal studies could track adults with varying food insecurity severity and insurance types in Jiangsu, Shandong and Sichuan for 1–3 years to verify causal directions, and include dynamic health outcome indicators (e.g., new chronic disease onset) to analyze the long-term impacts of food insecurity. Additionally, incorporating instrumental variable (IV) approaches to address endogeneity would be a fruitful direction for providing more robust evidence for policy interventions. Second, all health outcomes were self-reported by respondents, which may introduce recall bias and social desirability bias. This concern may be particularly relevant for stigmatized conditions such as mental disorders, where respondents may underreport diagnoses due to cultural stigma or privacy concerns. The absence of clinical verification or biomarker data may therefore limit the accuracy of our estimates. Future studies could combine survey data with medical records or biomarker measurements to improve measurement validity. Third, our sample likely underrepresented socioeconomically disadvantaged groups, particularly rural residents with limited Internet access and the extremely poor population, leading to downward estimation bias. These underrepresented groups typically face more severe food insecurity, which may further amplify the bias in the effect estimation of our study. Moreover, our findings should not be generalized to working-age adults without network access and other age/geographic groups beyond the sampled ones. In addition, the sample was restricted to individuals with a monthly income below ¥10,000 RMB. While this focused our analysis on populations most vulnerable to food insecurity, it introduces selection bias and limits the generalizability of our findings to higher-income groups. In wealthier populations, the moderating effect of health insurance might be less pronounced due to greater financial resilience, and the mediating role of healthcare accessibility could differ as their healthcare-seeking behaviors are less constrained by immediate costs. Consequently, the observed relationships between food insecurity, insurance, and health may not represent the full income spectrum. Finally, our results were prone to omitted variable bias, though our sensitivity checks ensured that the main findings were largely robust to unobserved confounding.

## 5 Conclusion

This study examined the relationship between household food insecurity and individual health among working-age adults in three Chinese provinces. Our findings revealed strong graded associations between food insecurity and a broad range of health outcomes, moderated by health insurance participation and mediated by healthcare accessibility. Policies enhancing health insurance coverage and access to healthcare services may reduce food insecurity and promote population health.

## Data Availability

The raw data supporting the conclusions of this article will be made available by the authors, without undue reservation.
