# Supplementary material for: Health insurance participation may mitigate the health impact of food insecurity among Chinese working-age adults
Source: Front Public Health. 2026 Apr 20;14:1808940. doi: 10.3389/fpubh.2026.1808940 (PMC13136130; doi:10.3389/fpubh.2026.1808940)
Supplement: Supplementary file 1 [file Supplementary_file_1.docx]

Supplementary Material

# 2 English Version questionnaires

**China Family Economic and Health Survey, 2024**

The purpose of this questionnaire is to investigate the economic status, material living conditions and physical and mental health of Chinese families, and the research results will promote understanding of related topics.

The questionnaire consists of 90 multiple choice questions covering five parts: demographic background information, food consumption, family economic status, family daily expenses, and personal health status. **It is expected to take 8-15 minutes to complete the questionnaire.**

Questionnaire serial number (code): _______ Date of data collection: ________

Interview district: ______

Data collector’s Name: ______________________

Supervisor’s Name: _________________________

**PART 1 Demographic background information**

1. Age:

[ ] 18-30

[ ] 31-35

[ ] 36-40

[ ] 41-45

[ ] 46-50

[ ] 51-55

[ ] 56-60

[ ] 61-65

[ ] 66-70

[ ] 71-75

[ ] 76-80

[ ] Above 80

2. Sex:

[ ] Male

[ ] Female

3. Residence:

[ ] Urban

[ ] Rural

4. Ethnicity:

[ ] Han

[ ] Minority

5. Province:

[ ] Jiangsu

[ ] Shandong

[ ] Sichuan

6. Highest education level:

[ ] Primary school graduate

[ ] Junior high school graduate

[ ] High school/secondary school/vocational school graduate

[ ] College/university graduate

[ ] Master/Doctor graduate

7. Marital status:

[ ] Unmarried

[ ] Married

[ ] Divorced

[ ] Widowed

[ ] Separated

8. How many children under the age of 18 live in your home?

[ ] No children

[ ] 1 child

[ ] 2 children

[ ] 3 children

[ ] 4 or more children

9. How many adults aged 18 and above (including you) live in your home?

[ ] 1 adult

[ ] 2 adults

[ ] 3 adults

[ ] 4 adults

[ ] 5 adults

[ ] 6 or more adults

**PART 2 Food consumption**

1. Do you think your daily diet is generally healthy?

[ ] Very healthy

[ ] Relatively healthy

[ ] General health

[ ] It's less healthy

[ ] Very unhealthy

[ ] I don't know

2. What do you think about the price of healthy food in general?

[ ] Very pricey

[ ] Rather pricey

[ ] Reasonable

[ ] Relatively cheap

[ ] Very cheap

[ ] I don't know

3. What do you think about the overall process of getting healthy food?

[ ] Very convenient

[ ] Relatively convenient

[ ] Generally convenient

[ ] Relatively inconvenient

[ ] Very inconvenient

[ ] I don't know

4. Of the following cooking methods, which ones do you usually use or eat? (Multiple options available)

[ ] Fry

[ ] Pickling

[ ] Cooking

[ ] Smoke and bake

[ ] Fry quickly

[ ] Cold sauce

[ ] Braising

[ ] Other methods

5. Of the following staples, which do you eat most often? (Multiple options available)

[ ] Milled rice (white rice/white rice porridge/rice noodles/glutinous rice)

[ ] Fine noodles (white steamed bread/noodles/bread/dumplings/rolls/pancakes)

[ ] Whole grains (whole wheat steamed bread/noodles/bread/whole grains/multigrains/brown rice/oats)

[ ] Corn (corn/corn rice/corn flour)

[ ] Roots (potatoes/sweet potatoes/yams/taro)

[ ] Other staple foods

6. Of the following cooking methods, which do you think is more healthful overall? (Multiple options available)

[ ] Fry

[ ] Pickling

[ ] Cooking

[ ] Smoke and bake

[ ] Fry quickly

[ ] Cold sauce

[ ] Braising

[ ] Other methods

[ ] I don't know

7. Of the following staple foods, which of the following do you think is more healthful overall? (Multiple options available)

[ ] Milled rice (white rice/white rice porridge/rice noodles/glutinous rice)

[ ] Fine noodles (white steamed bread/noodles/bread/dumplings/rolls/pancakes)

[ ] Whole grains (whole wheat steamed bread/noodles/bread/whole grains/multigrains/brown rice/oats)

[ ] Corn (corn/corn rice/corn flour)

[ ] Roots (potatoes/sweet potatoes/yams/taro)

[ ] Other staple foods

[ ] I don't know

**Here are 4 questions about food allergies in you and your family:**

8. Do you and your family have any food allergies? (Multiple options available)

[ ] No one has a food allergy

[ ] I have a food allergy

[ ] Other adults in the family have food allergies

[ ] Food allergies in children under the age of 3

[ ] Food allergy in children aged 3-17 years

9. If someone in your family has a food allergy, what are the main allergies? (Multiple options available)

[ ] Egg

[ ] Milk

[ ] Fish

[ ] Shellfish

[ ] soybean

[ ] Wheat

[ ] Peanut

[ ] Nuts

[ ] Sesame

[ ] Mango

[ ] Shrimp

[ ] Crab

[ ] Beef and mutton

[ ] Other allergens

[ ] No one has any food allergy

10. If someone in the family has a food allergy, what are the measures to be taken? (Multiple options available)

[ ] Avoiding allergens (not eating or touching allergenic foods)

[ ] Medication (such as taking antihistamines or anti-leukotriene antagonists)

[ ] Hormone therapy (e.g. adrenalin/corticosteroid injections)

[ ] Desensitization therapy (e.g. sublingual/subcutaneous immunotherapy)

[ ] Nutritional treatment (such as eating alternative formulas or foods to supplement nutrient intake)

[ ] Other countermeasures

[ ] No one has a food allergy

11. If someone in your family has a food allergy, in what ways has the food allergy affected your life and that of your family? (Multiple options available)

[ ] Limited dietary choices

[ ] Increased economic burden

[ ] Limited social activities

[ ] Occupational specialization is limited

[ ] Family tensions

[ ] Change of living environment and lifestyle

[ ] Other effects

[ ] No one has a food allergy

**Here are 8 questions about food consumption for you and your family:**

12. In the past 12 months, have you ever worried about not having enough food because of lack of money or other resources?

[ ] Yes

[ ] No

13. In the past 12 months, have you been unable to enjoy healthy and nutritious food due to lack of money or other resources?

[ ] Yes

[ ] No

14. In the past 12 months, have you had to eat only a few types of food due to lack of money or other resources?

[ ] Yes

[ ] No

15. In the past 12 months, have you skipped a meal because you did not have enough money or other resources to get food?

[ ] Yes

[ ] No

16. In the past 12 months, have you been unable to eat the amount of food you think you should because of lack of money or other resources?

[ ] Yes

[ ] No

17. In the past 12 months, has your family been deprived of food due to lack of money or other resources?

[ ] Yes

[ ] No

18. In the past 12 months, have you been hungry but did not eat because you did not have enough money or other resources to get food?

[ ] Yes

[ ] No

19. In the past 12 months, have you ever gone without food an entire day because of lack of money or other resources?

[ ] Yes

[ ] No

**PART 3 Family economic status**

1. What is your current employment status?

[ ] Have a full-time job

[ ] Have a part-time job

[ ] Have both full-time and part-time jobs

[ ] Unemployed

2. If you currently have a job, what is the nature of your main work?

[ ] Self-employed, without employees

[ ] Employers, with employees

[ ] Regular employees with long-term contracts

[ ] Temporary employees, with short-term contracts

[ ] Temporary employee, no contract

[ ] Currently unemployed

3. If you are currently unemployed, what is the main reason for not having a job?

[ ] Looking for a job

[ ] Family reasons

[ ] Health reasons

[ ] Student

[ ] Retire

[ ] Other reasons

[ ] Currently employed

4. What is your main occupation?

[ ] Uber driver, taxi driver

[ ] Truck driver, bus driver, private driver

[ ] Delivery man, delivery man

[ ] Sales staff, insurance consultants, real estate brokers, network with cargo owners broadcast

[ ] Security guard, doorman

[ ] Domestic staff, cleaning staff, waiter, babysitter

[ ] Freelancers (questionnaire fillers, customer service, etc.)

[ ] Company/store owner (physical or online)

[ ] Professional/technical worker (doctor, teacher, lawyer, engineer, editor, designer, etc.)

[ ] Managers/administrative officers/manager (factory directors, general manager, government officials, etc.)

[ ] Worker (foreman, workshop monitor, ordinary worker, etc.)

[ ] Office staff (secretary, clerk)

[ ] Farmer, fisherman, hunter

[ ] Military, police

[ ] Athlete, actor, performer

[ ] Other professions

[ ] Currently not working

5. What kind of organization do you work for?

[ ] Government agencies

[ ] Public institutions

[ ] State-owned enterprises

[ ] Private enterprise (Chinese-owned)

[ ] Private enterprise (owned by foreign companies or joint venture)

[ ] Collective enterprise

[ ] Household contract agriculture

[ ] Other types

[ ] Currently not working

6. What do you do on the side?

[ ] Uber driver, taxi driver

[ ] Truck driver, bus driver, private driver

[ ] Delivery man, delivery man

[ ] Sales staff, insurance consultants, real estate broker, network with cargo owners broadcast

[ ] Security guard, doorman

[ ] Domestic staff, cleaning staff, waiters, babysitters

[ ] Freelancers (questionnaire fillers, customer service, etc.)

[ ] Company/store owner (physical or online)

[ ] Professional/technical workers (doctors, teachers, lawyers, engineers, editors, designers, etc.)

[ ] Managers/administrative officers/managers (factory directors, general managers, government officials, etc.)

[ ] Workers (foreman, workshop monitor, ordinary worker, etc.)

[ ] Office staff (secretary, clerk)

[ ] Farmers, fishermen, hunters

[ ] Military, police

[ ] Athletes, actors, performers

[ ] Other side businesses

[ ] Currently no part-time side business

7. How many days do you work in an average week?

[ ] Does not work

[ ] 1 day

[ ] 2 days

[ ] 3 days

[ ] 4 days

[ ] 5 days

[ ] 6 days

[ ] 7 days

8. All together, how many hours do you work on average per day?

[ ] Does not work

[ ] 1-4 hours

[ ] 5 hours

[ ] 6 hours

[ ] 7 hours

[ ] 8 hours

[ ] 9 hours

[ ] 10 hours

[ ] 11 hours

[ ] 12 hours

[ ] More than 12 hours

9. What type of health insurance do you have? (Multiple options available)

[ ] the Urban Employee Basic Medical Insurance

[ ] the Urban and Rural Resident Basic Medical Insurance (the merger of the Urban Resident Basic Medical Insurance and the New Rural Cooperative Medical Care System)

[ ] the Urban Resident Basic Medical Insurance

[ ] the New Rural Cooperative Medical Care System

[ ] Huimin Insurance

[ ] Socialized medicine

[ ] Commercial health insurance

[ ] Employer-purchased health insurance

[ ] Other health insurance

[ ] Do not have health insurance, but would like to be covered

[ ] No health insurance, because voluntarily gave up

10. What type of endowment insurance do you have? (Multiple options available)

[ ] the Urban Employee Basic Endowment Insurance

[ ] the Urban and Rural Resident Basic Endowment Insurance (the merger of the Urban Resident Basic Endowment Insurance and the New Rural Cooperative Medical Care System)

[ ] the Urban Resident Basic Endowment Insurance

[ ] the New Rural Cooperative Medical Care System

[ ] Occupational annuity (for public institutions)

[ ] Enterprise annuity (for enterprises)

[ ] Personal pension

[ ] Commercial pension

[ ] Other pension insurance

[ ] No pension insurance, but want to participate in the insurance

[ ] No pension insurance, because voluntarily gave up

11. What other type of social security do you have? (multiple choices)

[ ] Unemployment insurance

[ ] Injury insurance

[ ] Maternity insurance

[ ] Housing fund

[ ] No social security above

12. In the past 12 months, what was your average monthly income (e.g. salary, bonus, commission, pension, etc.)? If unsure, provide a valuation.

[ ] No income

[ ] 1-500 yuan

[ ] 501-1000 yuan

[ ] 1001-2000 yuan

[ ] 2001-3000 yuan

[ ] 3001-4000 yuan

[ ] 4001-5000 yuan

[ ] 5001-6000 yuan

[ ] 6001-7000 yuan

[ ] 7001-8000 yuan

[ ] 8001-9000 yuan

[ ] 9001-10000 yuan

[ ] Above 10000 yuan

[ ] I don’t know

13. In the past 12 months, what was the average monthly income of you and your family (salary, bonus, commission, pension, etc.)? If unsure, provide a valuation.

[ ] No income

[ ] 1-500 yuan

[ ] 501-1000 yuan

[ ] 1001-2000 yuan

[ ] 2001-3000 yuan

[ ] 3001-4000 yuan

[ ] 4001-5000 yuan

[ ] 5001-6000 yuan

[ ] 6001-7000 yuan

[ ] 7001-8000 yuan

[ ] 8001-9000 yuan

[ ] 9001-10000 yuan

[ ] 10001-12000 yuan

[ ] 12001-15000 yuan

[ ] 15001-20000 yuan

[ ] Above 20000 yuan

[ ] I don’t know

14. How many people in your family have a steady monthly income?

[ ] No one's income is stable

[ ] 1 people

[ ] 2 people

[ ] 3 people

[ ] More than 3 people

15. What level do you think your family's economic conditions are in your local area?

[ ] Very good

[ ] Relatively good

[ ] Normal

[ ] Relatively poor

[ ] Very poor

16. Which of the following is your main housing type?

[ ] Home ownership

[ ] Rent public housing

[ ] Rent a private house

[ ] Unit welfare housing

[ ] Other houses

17. What is the gross area of your main apartment in square meters?

[ ] 1-30 square meters

[ ] 31-60 square meters

[ ] 61-100 square meters

[ ] 101-150 square meters

[ ] More than 150 square meters

18. How many times have you moved in the past 12 months?

[ ] Never moved

[ ] 1 time

[ ] 2 times

[ ] 3 times

[ ] More than 3 times

19. Which of the following problems exist in your current living environment? (Multiple options available)

[ ] Inadequate housing space (overcrowding, low privacy)

[ ] Insufficient ventilation and lighting (poor air mobility, dark light)

[ ] Noise and light pollution (excessive noise, noise and light)

[ ] Aging of building structure (wall cracking, pipe leakage, wall mold and fall off)

[ ] Inadequate infrastructure (unstable or lacking water/electricity/heating/gas/broadband/elevators)

[ ] Poor environmental hygiene (more public health problems such as garbage, sewage, pests)

[ ] Residential insecurity (fire hazards, public security problems)

[ ] Inconvenient transportation (far from subway stations, bus stops)

[ ] Lack of educational resources (far from quality schools, kindergartens)

[ ] Inadequate medical facilities (far from quality clinics, hospitals)

[ ] Inadequate community services (lack of community fitness venues, cultural activity centers)

[ ] Neighborhood tensions (neighbors are prone to sharp conflicts)

[ ] Other living environment problems

[ ] No living environment problems were found

20. Which of the following means of transportation does your home have? (multiple choices)

[ ] Automobile

[ ] Motorcycle

[ ] Electric moped/bicycle/tricycle

[ ] Other means of transportation

[ ] No means of transportation

21. Which of the following electronic devices are in your home? (multiple choices)

[ ] Computer (Desktop/Laptop/Tablet)

[ ] Smart phone

[ ] Color television

[ ] Other electronic devices

[ ] No electronic devices

22. What is the current total value of all the fixed assets owned by your family, including houses/apartments/vehicles/appliances/furniture/collectibles/land, etc. which are not in default? If unsure, provide a valuation.

[ ] Less than 10000 yuan

[ ] 10000-40000 yuan

[ ] 50000-90000 yuan

[ ] 100000-190000 yuan

[ ] 200000-490000 yuan

[ ] 500000-990000 yuan

[ ] 1-2 million yuan

[ ] More than 2 million yuan

[ ] I don't know

23. What is the current total value of all the liquid assets owned by your family, including cash/deposits/stocks/bonds/wealth management products? If unsure, provide a valuation.

[ ] Less than 1000 yuan

[ ] 1000-4999 yuan

[ ] 5000-9999 yuan

[ ] 10000-20000 yuan

[ ] 30000-50000 yuan

[ ] 60000-90000 yuan

[ ] 100000-200000 yuan

[ ] More than 200000 yuan

[ ] I don't know

24. What is the current total value of your family's debts, including mortgage/car loan/credit card loan/student loan/Internet loan/relative loan, etc.? If unsure, provide a valuation.

[ ] No debt

[ ] 1-999 yuan

[ ] 1000-4999 yuan

[ ] 5000-9999 yuan

[ ] 10000-20000 yuan

[ ] 30000-50000 yuan

[ ] 60000-90000 yuan

[ ] 100000-190000 yuan

[ ] 200000-490000 yuan

[ ] 500000-990000 yuan

[ ] 1-2 million yuan

[ ] More than 2 million yuan

[ ] I don't know

**PART 4 Family daily expenses**

1. During the past 12 months, what was the average monthly total living expenses of your family? If unsure, provide a valuation.

[ ] 0yuan

[ ] 1-500 yuan

[ ] 501-1000 yuan

[ ] 1001-2000 yuan

[ ] 2001-3000 yuan

[ ] 3001-4000 yuan

[ ] 4001-5000 yuan

[ ] 5001-6000 yuan

[ ] 6001-7000 yuan

[ ] 7001-8000 yuan

[ ] 8001-9000 yuan

[ ] 9001-10000 yuan

[ ] 10001-12000 yuan

[ ] 12001-15000 yuan

[ ] 15001-20000 yuan

[ ] Above 20000 yuan

[ ] I don’t know

2. In the past 12 months, what was your average monthly living expenses (such as rent, mortgage, etc.)? If unsure, provide a valuation.

[ ] 0yuan

[ ] 1-500 yuan

[ ] 501-1000 yuan

[ ] 1001-2000 yuan

[ ] 2001-3000 yuan

[ ] 3001-4000 yuan

[ ] 4001-5000 yuan

[ ] 5001-6000 yuan

[ ] 6001-7000 yuan

[ ] 7001-8000 yuan

[ ] 8001-9000 yuan

[ ] 9001-10000 yuan

[ ] 10001-12000 yuan

[ ] 12001-15000 yuan

[ ] 15001-20000 yuan

[ ] Above 20000 yuan

[ ] I don’t know

3. In the past 12 months, how much did your family spend on food per month on average (including groceries, take-out, restaurants, etc.)? If unsure, provide a valuation.

[ ] 0 yuan

[ ] 1-500 yuan

[ ] 501-1000 yuan

[ ] 1001-2000 yuan

[ ] 2001-3000 yuan

[ ] 3001-4000 yuan

[ ] 4001-5000 yuan

[ ] 5001-6000 yuan

[ ] 6001-7000 yuan

[ ] 7001-8000 yuan

[ ] 8001-9000 yuan

[ ] 9001-10000 yuan

[ ] 10001-12000 yuan

[ ] 12001-15000 yuan

[ ] 15001-20000 yuan

[ ] Above 20000 yuan

[ ] I don’t know

4. In the past 12 months, how much was your family's average monthly health care expenditure (including out-of-pocket expenses for medicine, hospitalization and surgery, medical insurance, etc.)? If unsure, provide a valuation.

[ ] 0 yuan

[ ] 1-500 yuan

[ ] 501-1000 yuan

[ ] 1001-2000 yuan

[ ] 2001-3000 yuan

[ ] 3001-4000 yuan

[ ] 4001-5000 yuan

[ ] 5001-6000 yuan

[ ] 6001-7000 yuan

[ ] 7001-8000 yuan

[ ] 8001-9000 yuan

[ ] 9001-10000 yuan

[ ] 10001-12000 yuan

[ ] 12001-15000 yuan

[ ] 15001-20000 yuan

[ ] Above 20000 yuan

[ ] I don’t know

5. In the past 12 months, how difficult has it been for your family to cover the daily expenses?

[ ] Very difficult

[ ] Some difficulties

[ ] Not too difficult

[ ] Not difficult at all

6. During the past 12 months, has your family experienced any of the following for financial reasons? (multiple choices)

[ ] Failure to pay rent or mortgage on time

[ ] Failure to repay loans other than the mortgage on time

[ ] Failure to pay educational expenses such as tuition and fees on time

[ ] Can not pay water, electricity, gas, mobile phone, Internet and other living expenses on time

[ ] Unable to buy food in time

[ ] Can not buy clothes, transportation and other necessities of life in time

[ ] Failure to seek medical attention or purchase medicines in a timely manner

[ ] Other situations where changes in initial consumption plans are forced

[ ] Have not experienced any of the above

7. In the past 12 months, has your family experienced any of the following situations in order to pay for daily household expenses? (multiple choices)

[ ] Increase income by working overtime, doing odd jobs, developing side businesses, etc

[ ] Use savings, provident fund and other personal savings to fill the household

[ ] Borrow from formal financial institutions such as banks

[ ] Borrow from informal financial institutions such as underground banks

[ ] Obtain financial assistance such as loans from relatives and friends

[ ] Donations of food or other goods from charities

[ ] Get cash donations from charities

[ ] Obtain cash by selling houses, selling cars, reselling items, etc

[ ] Apply to the government for social security subsidies such as subsistence allowance and unemployment insurance

[ ] Cut back on travel, entertainment, dining out and other non-essentials

[ ] Cut back on food spending

[ ] Cut back on necessities like medicine and rent

[ ] Cut costs by growing vegetable, fishing, mending clothes, etc

[ ] Postpone or cancel birth plans

[ ] Have not experienced any of the above

**PART 5 Personal health status**

1. How do you think of your physical health now?

[ ] Very good

[ ] Good

[ ] Average

[ ] Poor

[ ] Very poor

2. What do you think of your mental health status now?

[ ] Very good

[ ] Good

[ ] Average

[ ] Poor

[ ] Very poor

3. Have you been diagnosed with any of the following chronic diseases? (multiple choices)

[ ] Hypertension

[ ] Hyperlipemia

[ ] Hyperglycemia

[ ] Diabetes

[ ] Nutritional deficiency

[ ] Anemia

[ ] Gout/high uric acid

[ ] Osteoporosis

[ ] Disc disease

[ ] Tumor/cancer

[ ] Cerebrovascular diseases (such as cerebral stroke)

[ ] Heart disease (e.g. chronic heart failure/coronary heart disease/myocardial infarction)

[ ] Respiratory diseases (e.g. COPD/asthma)

[ ] Digestive diseases (e.g. chronic gastroenteritis/hepatitis/pancreatitis/cholecystitis)

[ ] Urinary diseases (such as chronic nephritis/renal failure)

[ ] Diseases of the endocrine system (e.g. hyperthyroidism/hypothyroidism)

[ ] Rheumatic diseases (e.g. rheumatoid arthritis/lupus erythematosus)

[ ] Neurological diseases (e.g. Multiple sclerosis/Alzheimer's/Parkinson's)

[ ] Other chronic diseases

[ ] No chronic disease diagnosis

4. Have you been diagnosed with any of the following mental illnesses? (multiple choices)

[ ] Depression

[ ] Anxiety disorder

[ ] Mania/bipolar disorder

[ ] Insomnia

[ ] Obsessive compulsive disorder

[ ] Schizophrenia

[ ] Autism/autism

[ ] Personality disorder

[ ] Anorexia

[ ] Other mental diseases

[ ] No diagnosis of mental illness

5. In the past 12 months, have you been treated for an injury or illness? (Multiple options available)

[ ] Yes, due to chronic diseases (e.g. diabetes)

[ ] Yes, due to acute illnesses (e.g. influenza)

[ ] Yes, due to mental disorders (e.g. depression)

[ ] Yes, due to accidental injuries (e.g. fall)

[ ] Yes, due to intentional injuries (e.g. self-injury or other injury)

[ ] No, no medical treatment for injury

6. During the past 12 months, have you often suffered from pain in any part of your body?

[ ] Not at all

[ ] Occasionally

[ ] Sometimes

[ ] Quite often

[ ] All the time

7. In the past 12 months, have you had any of the following disabilities for a week or more? (Multiple options available)

[ ] Visual disability

[ ] Hearing and speech disability

[ ] Intellectual disability

[ ] Physical disability

[ ] Mental disability

[ ] Without any disability

8. In the past 12 months, have you or your family members been unable to work or do household chores due to disability or health reasons? (Multiple options available)

[ ] Yes, I myself have been unable to work or do housework properly due to disability or health reasons

[ ] Yes, my family members have been unable to work or do housework due to disability or health reasons

[ ] No, my family and I have been able to work normally and do housework

[ ] No, my family and I have been unable to work or do housework properly due to other (non-disability or health) reasons

9. Which of the following health services have you used in the past 12 months? If used, once or many times? (Multiple options available)

**If you select "Did not use health services", you do not need to answer question 10.**

[ ] Physical examination (single)

[ ] Physical examination (multiple times)

[ ] General clinic (single visit)

[ ] General clinic (multiple times)

[ ] Emergency room (single visit)

[ ] Emergency room (multiple times)

[ ] In-patient hospitalization (single)

[ ] In-patient hospitalization (multiple times)

[ ] Surgery (single)

[ ] Surgery (multiple times)

[ ] Did not use health services

10. In the past 12 months, what percentage of your personal medical expenses (including doctor visits, medicine, hospitalization, surgery, etc.) were out-of-pocket expenses (the percentage not covered by insurance or other means)?

[] 0% (full reimbursement or free of charge)

[ ] 1-10%

[ ] 11-20%

[ ] 21-30%

[ ] 31-40%

[ ] 41-50%

[ ] 51-60%

[ ] 61-70%

[ ] 71-80%

[ ] 81-90%

[ ] 91-99%

[ ] 100% (full expense)

11. Do you smoke (including cigarettes, e-cigarettes, pipes, tobacco chews, etc.)?

[ ] Never smoked

[ ] Already quit smoking

[ ] Currently smoking

12. In the past 12 months, how often did you drink alcohol (including beer, wine, wine, etc.)?

[ ] Never

[ ] Less than once a month

[ ] Once or twice a month

[ ] Once or twice a week

[ ] 3-4 times a week

[ ] Almost every day

13. How long do you sit per day on average (work, eat, play, rest, etc.)?

[ ] 1-3 hours

[ ] 4-6 hours

[ ] 7-9 hours

[ ] More than 9 hours

14. How often do you usually engage in moderate or intensive exercise or recreational activities (each lasting at least 10 minutes and causing rapid breathing)?

[ ] Every day

[ ] 4-6 times a week

[ ] 1-3 times a week

[ ] 1-3 times a month

[ ] Almost no exercise due to external limitations

[ ] Almost no exercise due to personal preferences

15. Do you have an overall routine? (Multiple options available)

[ ] Sleep and wake up at irregular times

[ ] Dinner times are irregular

[ ] Irregular commuting hours

[ ] The general rule of life and rest time

16. How would you describe your weight?

[ ] Slender

[ ] Normal

[ ] Overweight

[ ] Obesity

17. How unhealthy do you think your diet is overall?

[ ] Very unhealthy

[ ] Somewhat unhealthy

[ ] Not healthy or unhealthy

[ ] Somewhat healthy

[ ] Very healthy

[ ] I don't know

18. During the past two weeks, have you often felt down, depressed, or hopeless?

[ ] No

[ ] For a few days

[ ] More than half of the days

[ ] Almost every day

19. In the past two weeks, have you often lost interest or enjoyment in something that normally interests you?

[ ] No

[ ] For a few days

[ ] More than half of the days

[ ] Almost every day

20. During the past two weeks, have you often felt nervous, anxious, or on edge?

[ ] No

[ ] For a few days

[ ] More than half of the days

[ ] Almost every day

21. During the past two weeks, have you often felt unable to control or stop worrying?

[ ] No

[ ] For a few days

[ ] More than half of the days

[ ] Almost every day

22. How is your sleep?

[ ] Very poor

[ ] Poor

[ ] Average

[ ] Good

[ ] Very good

23. Do you often feel lonely?

[ ] Always

[ ] Often

[ ] Sometimes

[ ] Rarely

[ ] Never

24. Is there someone around who can help you when you need it?

[ ] Always

[ ] Often

[ ] Sometimes

[ ] Occasionally

[ ] Never

25. Overall, how satisfied are you with your life?

[ ] Extremely satisfied

[ ] Very satisfied

[ ] Satisfied

[ ] Not very satisfied

[ ] Not at all satisfied

26. In the past 12 months, have you made any comments or suggestions on public issues such as health, security, etc. in your community through the neighborhood committee, property management committee, or owner committee?

[ ] Never proposed

[ ] Occasionally raised

[ ] Often proposed

**Here are 4 questions about the use of digital devices:**

27. Does your home have any of the following digital devices that can be connected to the Internet? (multiple choices)

[ ] Smart phone

[ ] Desktop computer

[ ] Laptop

[ ] Tablet computer

[ ] Other electronic devices

28. In the past 12 months, which of the following activities have you done via the Internet? (multiple choices)

[ ] Communication

[ ] Entertainment

[ ] Shopping

[ ] Study

[ ] Work

[ ] do business

[ ] Financial management

[ ] None of the above

29. Which of the following Internet health services have you used in the past 12 months? (Multiple options available)

[ ] Online booking

[ ] Remote consultation

[ ] Drug purchase

[ ] Health management

[ ] Online inquiry

[ ] Other Internet health services

[ ] Not using Internet health services

30. What do you think of your skills with digital devices?

[ ] Very skilled

[ ] Somewhat skilled

[ ] Average

[ ] Not very skilled

[ ] Not at all skilled

31. How good do you think you are at detecting false information on the Internet?

[ ] Very good

[ ] Good

[ ] Average

[ ] Poor

[ ] Very poor
