# Supplementary material for: Health insurance participation may mitigate the health impact of food insecurity among Chinese working-age adults
Source: Front Public Health. 2026 Apr 20;14:1808940. doi: 10.3389/fpubh.2026.1808940 (PMC13136130; doi:10.3389/fpubh.2026.1808940)
Supplement: Supplementary file 2 [file Table_1.docx]

Supplementary Material

# Supplementary Figures

**Supplementary Table A**

The Food Insecurity Experience Scale Survey Module

|  | **Standard label** | **Question** |
| --- | --- | --- |
| **1** | **WORRIED** | During the last 12 MONTHS, was there a time when You were worried you would not have enough food to eat because of a lack of money or other resources? |
| **2** | **HEALTHY** | Still thinking about the last 12 MONTHS, was there a time when you were unable to eat healthy and nutritious food because of a lack of money or other resources? |
| **3** | **FEWFOODS** | Was there a time when you ate only a few kinds of foods because of a lack of money or other resources? |
| **4** | **SKIPPED** | Was there a time when you had to skip a meal because there was not enough money or other resources to get food? |
| **5** | **ATELESS** | Still thinking about the last 12 MONTHS, was there a time when you ate less than you thought you should because of a lack of money or other resources? |
| **6** | **RANOUT** | Was there a time when your household ran out of food because of a lack of money or other resources? |
| **7** | **HUNGRY** | Was there a time when you were hungry but did not eat because there was not enough money or other resources for food? |
| **8** | **WHOLEDAY** | During the last 12 MONTHS, was there a time when you went without eating for a whole day because of a lack of money or other resources? |

**Supplementary Table B**

The validity of The Food Insecurity Experience Scale

| **Items** | **Standardized factor loadings** | **SD** | **Z** | **P** |
| --- | --- | --- | --- | --- |
| **WORRIED** | 0.59 | 0.11 | 54.45 | <0.001 |
| **HEALTHY** | 0.54 | 0.12 | 46.54 | <0.001 |
| **FEWFOODS** | 0.52 | 0.01 | 43.18 | <0.001 |
| **SKIPPED** | 0.75 | 0.01 | 93.23 | <0.001 |
| **ATELESS** | 0.56 | 0.01 | 49.25 | <0.001 |
| **RANOUT** | 0.75 | 0.01 | 90.44 | <0.001 |
| **HUNGRY** | 0.80 | 0.01 | 109.76 | <0.001 |
| **WHOLEDAY** | 0.68 | 0.01 | 70.97 | <0.001 |
|  |  |  |  |  |
| **Cronbach’s α** | 0.86 |  |  |  |
| **CFI** | 0.98 |  |  |  |
| **TLI** | 0.97 |  |  |  |
| **RMSEA** | 0.07 |  |  |  |
| **SRMR** | 0.03 |  |  |  |

**Notes** SD = standard deviation.

P is the p-value for the t-test difference between the food-secure and food-insecure individuals.

**Supplementary Table C**

The comparison table for UEBMI and URRBMI in Nanjing, Jiangsu, China

| **Item** | **The Urban Employee Medical Insurance (UEBMI)** | **The Urban and Rural Resident Medical Insurance (URRBMI)** |
| --- | --- | --- |
| **Insured Population** | Employed workers, self-employed individuals, retirees, etc. | Residents who are not covered by the Urban Employee Medical Insurance |
| **Payment Method** | Monthly contributions, shared by both employers and individuals | Annual contributions, borne by individuals or families |
| **Payment Standard (2024)** | Employer contribution rate about 7%, individual contribution rate about 2% | Multiple tiers available, individual payment standards range from a few hundred to a few thousand yuan |
| **Coverage** | Inpatient care, outpatient major illness, general outpatient care, outpatient chronic diseases, etc. | Inpatient care, outpatient major illness, general outpatient care, outpatient chronic diseases, etc. |
| **Reimbursement Ratio** | Inpatient: 85%-95%, General Outpatient: 50%-70% | Inpatient: 75%-85%, General Outpatient: 50%-60% |
| **Deductible** | Inpatient: 800-1200 yuan, General Outpatient: varies by hundreds of yuan | Inpatient: varies from a few hundred to over a thousand yuan, General Outpatient: varies from tens to hundreds of yuan |
| **Ceiling** | Inpatient: several hundred thousand yuan; General outpatient: a few thousand to ten thousand yuan | Inpatient: over ten thousand yuan; General outpatient: a few thousand yuan |
| **Medical Treatment Abroad** | Requires registration for out-of-town medical treatment, reimbursement ratio slightly reduced | Requires registration for out-of-town medical treatment, reimbursement ratio slightly reduced |
| **Personal Account** | Personal account available for outpatient expenses and pharmacy purchases | Personal account available for some tiers, used for the same purposes as Urban Employee Medical Insurance |

**Notes** The information provided in this table can be obtained from ybj.nanjing.gov.cn

**Supplementary Table D**

The number of missing values in covariates

| **Missing values** | **Frequency** | **Percentage** |
| --- | --- | --- |
| Family income | 72 | 1.5% |
| Fixed assets | 255 | 5.32% |
| Liquid assets | 307 | 6.40% |
| Debt | 135 | 2.82% |

**Supplementary Table E**

Tests for stability and omitted variables bias (The Oster (2019) Test)

|  | **Oster bounds []** 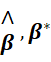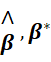 **( δ=1 )** | | | |
| --- | --- | --- | --- | --- |
| **Rmax** | 1.2 | 1.3 | 1.4 | 1.5 |
| **Any chronic condition** | [0.096,0.107] | [0.091,0.107] | [0.085,0.107] | [0.077,0.107] |
| **Any mental disorder** | [0.084,0.108] | [0.071,0.108] | [0.058,0.108] | [0.044,0.108] |
| **Any disability** | [0.055,0.075] | [0.048,0.075] | [0.038,0.075] | [0.027,0.075] |
| **Any doctor visit** | [0.095,0.101] | [0.091,0.101] | [0.086,0.101] | [0.082,0.101] |

**Supplementary Table F**

Descriptive statistics of outcome variables, overall and by food insecurity status

|  | **Total**  **(n=4795)** | | **Food secure (n=3399)** | | **Food insecure (n=1396)** | | **P** |
| --- | --- | --- | --- | --- | --- | --- | --- |
|  | **Mean** | **SD** | **Mean** | **SD** | **Mean** | **SD** |  |
| Chronic conditions | 0.45 | 0.50 | 0.39 | 0.49 | 0.60 | 0.49 | <0.001 |
| Mental disorders | 0.16 | 0.37 | 0.10 | 0.30 | 0.31 | 0.46 | <0.001 |
| Disabilities | 0.09 | 0.28 | 0.05 | 0.21 | 0.18 | 0.39 | <0.001 |
| Doctor visits | 0.36 | 0.48 | 0.31 | 0.46 | 0.48 | 0.50 | <0.001 |

**Notes** SD = standard deviation.

P is the p-value for the t-test difference between the food-secure and food-insecure individuals.

**Supplementary Table G**

The correlation between 4-category household food insecurity and health status (complete output)

|  | **(1)** | **(2)** | **(3)** | **(4)** |
| --- | --- | --- | --- | --- |
|  | **Any chronic condition** | **Any mental disorder** | **Any disability** | **Any doctor visit** |
| **Main**. | | | | |
| Food security | 1.00 | 1.00 | 1.00 | 1.00 |
|  | [1.00,1.00] | [1.00,1.00] | [1.00,1.00] | [1.00,1.00] |
| Mild food insecurity | 2.01*** | 2.22*** | 1.88** | 1.72*** |
|  | [1.50,2.69] | [1.53,3.21] | [1.17,3.02] | [1.29,2.27] |
| Moderate food insecurity | 3.82*** | 4.80*** | 4.42*** | 2.33*** |
|  | [2.29,6.38] | [2.94,7.85] | [2.52,7.78] | [1.45,3.74] |
| Severe food insecurity | 2.15* | 6.87*** | 7.39*** | 3.22*** |
|  | [1.10,4.18] | [3.65,12.93] | [3.48,15.67] | [1.63,6.36] |
| **Age** | | | | |
| 31-35 years old | 1.00 | 1.00 | 1.00 | 1.00 |
|  | [1.00,1.00] | [1.00,1.00] | [1.00,1.00] | [1.00,1.00] |
| 36-40 years old | 1.29** | 1.02 | 1.35 | 0.84* |
|  | [1.10,1.51] | [0.81,1.28] | [0.98,1.87] | [0.71,0.99] |
| 41-60 years old | 1.88*** | 0.90 | 2.07*** | 0.97 |
|  | [1.56,2.28] | [0.68,1.20] | [1.42,3.00] | [0.80,1.19] |
| **Sex** | | | | |
| female | 1.00 | 1.00 | 1.00 | 1.00 |
|  | [1.00,1.00] | [1.00,1.00] | [1.00,1.00] | [1.00,1.00] |
| male | 0.83 | 0.67* | 1.06 | 0.73* |
|  | [0.65,1.07] | [0.48,0.94] | [0.67,1.67] | [0.56,0.95] |
| **Residence** | | | | |
| Rural residence | 1.00 | 1.00 | 1.00 | 1.00 |
|  | [1.00,1.00] | [1.00,1.00] | [1.00,1.00] | [1.00,1.00] |
| Urban residence | 1.11 | 1.28 | 0.78 | 0.74* |
|  | [0.83,1.48] | [0.84,1.94] | [0.48,1.26] | [0.55,0.99] |
| **Ethnicity** | | | | |
| Han | 1.00 | 1.00 | 1.00 | 1.00 |
|  | [1.00,1.00] | [1.00,1.00] | [1.00,1.00] | [1.00,1.00] |
| Minority | 0.76 | 4.26** | 1.78 | 0.98 |
|  | [0.31,1.86] | [1.72,10.55] | [0.52,6.03] | [0.44,2.17] |
| **Province** | | | | |
| Jiangsu | 1.00 | 1.00 | 1.00 | 1.00 |
|  | [1.00,1.00] | [1.00,1.00] | [1.00,1.00] | [1.00,1.00] |
| Shandong | 1.17 | 1.00 | 1.13 | 0.86 |
|  | [0.92,1.48] | [0.71,1.40] | [0.72,1.75] | [0.67,1.10] |
| Sichuan | 1.07 | 1.01 | 0.98 | 1.01 |
|  | [0.82,1.39] | [0.70,1.46] | [0.60,1.59] | [0.77,1.33] |
| **Education** | | | | |
| Below junior high school | 1.00 | 1.00 | 1.00 | 1.00 |
|  | [1.00,1.00] | [1.00,1.00] | [1.00,1.00] | [1.00,1.00] |
| High school diploma | 0.90 | 5.28*** | 2.96** | 0.89 |
|  | [0.47,1.72] | [2.17,12.81] | [1.31,6.72] | [0.48,1.65] |
| College degree | 0.72 | 2.54* | 1.57 | 0.66 |
|  | [0.38,1.33] | [1.04,6.20] | [0.68,3.61] | [0.36,1.21] |
| Graduate degree | 0.70 | 4.13** | 2.14 | 0.75 |
|  | [0.36,1.39] | [1.60,10.62] | [0.84,5.43] | [0.39,1.44] |
| **Number of children** | | | | |
| No children | 1.00 | 1.00 | 1.00 | 1.00 |
|  | [1.00,1.00] | [1.00,1.00] | [1.00,1.00] | [1.00,1.00] |
| 1 child | 0.85 | 1.28 | 1.02 | 0.94 |
|  | [0.62,1.16] | [0.84,1.95] | [0.58,1.80] | [0.67,1.30] |
| 2 children | 0.68* | 1.36 | 1.34 | 0.79 |
|  | [0.47,0.97] | [0.84,2.21] | [0.68,2.63] | [0.54,1.15] |
| 3 or more children | 0.73 | 2.34* | 2.20 | 0.73 |
|  | [0.29,1.80] | [1.02,5.33] | [0.78,6.21] | [0.31,1.71] |
| **Marital status** | | | | |
| Unmarried | 1.00 | 1.00 | 1.00 | 1.00 |
|  | [1.00,1.00] | [1.00,1.00] | [1.00,1.00] | [1.00,1.00] |
| Married | 1.18 | 0.67 | 0.47* | 0.65* |
|  | [0.81,1.73] | [0.42,1.07] | [0.24,0.93] | [0.43,0.96] |
| Divorced/Separated/ Widowed | 0.94 | 0.82 | 0.96 | 0.71 |
|  | [0.50,1.76] | [0.36,1.88] | [0.34,2.68] | [0.38,1.33] |
| **Number of adults** | | | | |
| 1 adults | 1.00 | 1.00 | 1.00 | 1.00 |
|  | [1.00,1.00] | [1.00,1.00] | [1.00,1.00] | [1.00,1.00] |
| 2 adults | 0.81 | 1.17 | 1.02 | 1.40 |
|  | [0.52,1.25] | [0.65,2.12] | [0.48,2.18] | [0.86,2.29] |
| 3 adults | 0.91 | 0.77 | 0.80 | 1.43 |
|  | [0.58,1.44] | [0.43,1.40] | [0.37,1.74] | [0.86,2.36] |
| 4 or more adults | 1.10 | 0.86 | 1.15 | 1.73* |
|  | [0.68,1.77] | [0.45,1.64] | [0.51,2.58] | [1.03,2.91] |
| **Health insurance** | | | | |
| no health insurance | 1.00 | 1.00 | 1.00 | 1.00 |
|  | [1.00,1.00] | [1.00,1.00] | [1.00,1.00] | [1.00,1.00] |
| UEBMI | 2.66* | 2.06 | 0.79 | 2.88* |
|  | [1.21,5.87] | [0.89,4.77] | [0.27,2.31] | [1.26,6.57] |
| URRBMI | 1.84 | 2.67* | 1.10 | 2.74* |
|  | [0.83,4.11] | [1.13,6.30] | [0.36,3.32] | [1.18,6.33] |
| Only other types of health insurance | 1.48 | 2.71* | 1.83 | 2.60 |
|  | [0.57,3.88] | [1.02,7.22] | [0.39,8.62] | [0.97,7.00] |
| **Family income** | | | | |
| ¥0-4k | 1.00 | 1.00 | 1.00 | 1.00 |
|  | [1.00,1.00] | [1.00,1.00] | [1.00,1.00] | [1.00,1.00] |
| ¥4-9k | 1.48 | 0.52 | 0.59 | 0.96 |
|  | [0.82,2.68] | [0.27,1.03] | [0.30,1.15] | [0.54,1.71] |
| ¥9-15k | 1.25 | 0.37** | 0.24*** | 1.10 |
|  | [0.69,2.24] | [0.19,0.71] | [0.13,0.46] | [0.63,1.93] |
| Above ¥15k | 1.32 | 0.53 | 0.47* | 1.41 |
|  | [0.73,2.38] | [0.27,1.05] | [0.24,0.92] | [0.79,2.49] |
| Unknown | 0.71 | 0.89 | 0.10** | 0.38 |
|  | [0.22,2.30] | [0.22,3.55] | [0.02,0.42] | [0.11,1.33] |
| **Fixed assets** | | | | |
| ¥0-40k | 1.00 | 1.00 | 1.00 | 1.00 |
|  | [1.00,1.00] | [1.00,1.00] | [1.00,1.00] | [1.00,1.00] |
| ¥40-200k | 0.86 | 0.66 | 1.20 | 0.60 |
|  | [0.48,1.54] | [0.36,1.19] | [0.59,2.42] | [0.35,1.04] |
| ¥200-1000k | 0.75 | 0.46* | 1.25 | 0.55* |
|  | [0.42,1.33] | [0.25,0.86] | [0.55,2.84] | [0.32,0.97] |
| Above ¥1000k | 0.78 | 0.46* | 0.68 | 0.55* |
|  | [0.43,1.41] | [0.24,0.87] | [0.28,1.63] | [0.31,0.97] |
| Unknown | 0.74 | 0.35* | 2.25 | 0.34* |
|  | [0.32,1.67] | [0.12,1.00] | [0.60,8.44] | [0.14,0.84] |
| **Liquid assets** | | | | |
| Below ¥5k | 1.00 | 1.00 | 1.00 | 1.00 |
|  | [1.00,1.00] | [1.00,1.00] | [1.00,1.00] | [1.00,1.00] |
| ¥5-20k | 1.41 | 2.41** | 2.52* | 1.68 |
|  | [0.80,2.50] | [1.28,4.52] | [1.11,5.71] | [0.96,2.95] |
| ¥20-100k | 1.28 | 1.71 | 1.99 | 1.94* |
|  | [0.72,2.28] | [0.84,3.47] | [0.81,4.91] | [1.09,3.44] |
| Above ¥100k | 1.48 | 1.76 | 1.59 | 1.95* |
|  | [0.84,2.60] | [0.88,3.54] | [0.60,4.20] | [1.10,3.47] |
| Unknown | 1.26 | 4.17* | 0.90 | 2.06 |
|  | [0.58,2.73] | [1.39,12.44] | [0.20,4.01] | [0.87,4.88] |
| **Debts** | | | | |
| No debt | 1.00 | 1.00 | 1.00 | 1.00 |
|  | [1.00,1.00] | [1.00,1.00] | [1.00,1.00] | [1.00,1.00] |
| ¥1-10k | 1.56 | 2.18* | 2.13 | 1.92* |
|  | [0.90,2.72] | [1.15,4.13] | [0.94,4.82] | [1.15,3.23] |
| ¥10-100k | 1.63** | 1.56* | 1.92* | 1.62** |
|  | [1.16,2.31] | [1.03,2.38] | [1.09,3.38] | [1.15,2.28] |
| Above ¥100k | 1.28* | 1.45* | 1.28 | 1.48** |
|  | [1.01,1.63] | [1.03,2.05] | [0.76,2.14] | [1.15,1.89] |
| Unknown | 1.31 | 1.11 | 0.76 | 1.60 |
|  | [0.57,2.99] | [0.38,3.28] | [0.16,3.71] | [0.63,4.05] |
| **Smoking status** | | | | |
| Never smoked | 1.00 | 1.00 | 1.00 | 1.00 |
|  | [1.00,1.00] | [1.00,1.00] | [1.00,1.00] | [1.00,1.00] |
| Quit | 2.12*** | 2.29*** | 2.02** | 1.54** |
|  | [1.54,2.92] | [1.56,3.38] | [1.20,3.41] | [1.12,2.12] |
| Smoked | 1.76** | 1.41 | 1.25 | 1.43* |
|  | [1.24,2.50] | [0.93,2.13] | [0.71,2.20] | [1.01,2.01] |
| **Drinking frequency** | | | | |
| Do not drink | 1.00 | 1.00 | 1.00 | 1.00 |
|  | [1.00,1.00] | [1.00,1.00] | [1.00,1.00] | [1.00,1.00] |
| Less than once a month | 1.11 | 1.23 | 1.41 | 1.32 |
|  | [0.83,1.48] | [0.76,2.00] | [0.72,2.77] | [0.97,1.81] |
| Once or twice a month | 1.08 | 1.78* | 1.91* | 1.46* |
|  | [0.80,1.46] | [1.13,2.79] | [1.07,3.38] | [1.06,2.01] |
| More than once a week | 1.26 | 2.54*** | 2.37** | 1.62** |
|  | [0.89,1.79] | [1.61,4.01] | [1.23,4.54] | [1.13,2.33] |
| N | 4795 | 4795 | 4795 | 4795 |

**Notes** Adjusted odds ratios; 95% confidence intervals in brackets.

^*^ *p*<0.05, ^**^ *p*<0.01, ^***^ *p*<0.001

**Supplementary Table H**

The correlation between 3-category household food insecurity and health status

|  | **(1)** | **(2)** | **(3)** | **(4)** |
| --- | --- | --- | --- | --- |
|  | **Any chronic condition** | **Any mental disorder** | **Any disability** | **Any doctor visit** |
| Food security | 1.00 | 1.00 | 1.00 | 1.00 |
|  | [1.00,1.00] | [1.00,1.00] | [1.00,1.00] | [1.00,1.00] |
| Mild food insecurity | 2.01*** | 2.22*** | 1.88** | 1.72*** |
|  | [1.50,2.69] | [1.53,3.21] | [1.17,3.02] | [1.29,2.27] |
| Severe food insecurity | 3.22*** | 5.28*** | 5.12*** | 2.54*** |
|  | [2.10,4.93] | [3.45,8.07] | [3.08,8.52] | [1.68,3.83] |
| N | 4795 | 4795 | 4795 | 4795 |

**Notes** Adjusted odds ratios; 95% confidence intervals in brackets.

All models were weighted with post-stratification sampling weights and adjusted for age, sex, residence, ethnicity, province, education, number of children, marital status, number of adults, health insurance participation, family income, fixed assets, liquid assets, debt, smoking status, and drinking frequency.

^*^ *p* < 0.05, ^**^ *p* < 0.01, ^***^ *p* < 0.001

**Supplementary Table I**

The correlation between binary food insecurity Status (treating marginal food insecurity as food insecure) and health

|  | **(1)** | **(2)** | **(3)** | **(4)** |
| --- | --- | --- | --- | --- |
|  | **Any chronic condition** | **Any mental disorder** | **Any disability** | **Any doctor visit** |
| Food security | 1.00 | 1.00 | 1.00 | 1.00 |
|  | [1.00,1.00] | [1.00,1.00] | [1.00,1.00] | [1.00,1.00] |
| Food insecurity | 2.25*** | 2.90*** | 2.68*** | 1.89*** |
|  | [1.73,2.92] | [2.12,3.96] | [1.80,4.00] | [1.47,2.43] |
| N | 4795 | 4795 | 4795 | 4795 |

**Notes** Adjusted odds ratios; 95% confidence intervals in brackets.

All models were weighted with post-stratification sampling weights and adjusted for age, sex, residence, ethnicity, province, education, number of children, marital status, number of adults, health insurance participation, family income, fixed assets, liquid assets, debt, smoking status, and drinking frequency.

^*^ *p* < 0.05, ^**^ *p* < 0.01, ^***^ *p* < 0.001

**Supplementary Table J**

The correlation between binary food insecurity status (treating marginal food insecurity as food secure) and health

|  | **(1)** | **(2)** | **(3)** | **(4)** |
| --- | --- | --- | --- | --- |
|  | **Any chronic condition** | **Any mental disorder** | **Any disability** | **Any doctor visit** |
| Food security | 1.00 | 1.00 | 1.00 | 1.00 |
|  | [1.00,1.00] | [1.00,1.00] | [1.00,1.00] | [1.00,1.00] |
| Food insecurity | 2.48*** | 3.79*** | 3.91*** | 2.06*** |
|  | [1.63,3.78] | [2.52,5.71] | [2.42,6.30] | [1.37,3.10] |
| N | 4795 | 4795 | 4795 | 4795 |

**Notes** Adjusted odds ratios; 95% confidence intervals in brackets.

All models were weighted with post-stratification sampling weights and adjusted for age, sex, residence, ethnicity, province, education, number of children, marital status, number of adults, health insurance participation, family income, fixed assets, liquid assets, debt, smoking status, and drinking frequency.

^*^ *p* < 0.05, ^**^ *p* < 0.01, ^***^ *p* < 0.001

**Supplementary Table K**

The correlation between 4-category household food insecurity and multi morbidity (the negative binomial regression model)

|  | **(1)** | **(2)** | **(3)** | **(4)** |
| --- | --- | --- | --- | --- |
|  | **Multiple chronic conditions** | **Multiple mental disorders** | **Multiple disabilities** | **Multiple reasons for doctor visits** |
| Food security | 1.00 | 1.00 | 1.00 | 1.00 |
|  | [1.00,1.00] | [1.00,1.00] | [1.00,1.00] | [1.00,1.00] |
| Mild food insecurity | 1.46*** | 2.08*** | 1.82** | 1.38*** |
|  | [1.25,1.71] | [1.52,2.85] | [1.22,2.72] | [1.17,1.63] |
| Moderate food insecurity | 2.07*** | 3.32*** | 3.34*** | 2.03*** |
|  | [1.67,2.56] | [2.35,4.68] | [2.12,5.26] | [1.59,2.58] |
| Severe food insecurity | 2.54*** | 4.04*** | 5.83*** | 2.45*** |
|  | [1.79,3.61] | [2.68,6.09] | [3.63,9.38] | [1.79,3.35] |
| lnalpha | 0.43*** | 1.56 | 0.35 | 0.00 |
|  | [0.32,0.58] | [1.00,2.46] | [0.07,1.76] | [0.00,0.00] |
| N | 4795 | 4795 | 4795 | 4795 |

**Notes** Incidence rate ratio; 95% confidence intervals in brackets

All models were weighted with post-stratification sampling weights and adjusted for age, sex, residence, ethnicity, province, education, number of children, marital status, number of adults, health insurance participation, family income, fixed assets, liquid assets, debt, smoking status, and drinking frequency.

^*^ *p* < 0.05, ^**^ *p* < 0.01, ^***^ *p* < 0.001

**Supplementary Table L**

The correlation between 4-category household food insecurity and multi morbidity (the zero-inflated Possion model)

|  | **(1)** | **(2)** | **(3)** | **(4)** |
| --- | --- | --- | --- | --- |
|  | **Multiple chronic conditions** | **Multiple mental disorders** | **Multiple disabilities** | **Multiple reasons for doctor visits** |
| Food security | 1.00 | 1.00 | 1.00 | 1.00 |
|  | [1.00,1.00] | [1.00,1.00] | [1.00,1.00] | [1.00,1.00] |
| Mild food insecurity | 1.19** | 1.01 | 1.41 | 1.52*** |
|  | [1.06,1.33] | [0.80,1.27] | [0.95,2.10] | [1.36,1.70] |
| Moderate food insecurity | 1.60*** | 1.15 | 1.56* | 1.89*** |
|  | [1.38,1.85] | [0.89,1.49] | [1.03,2.37] | [1.61,2.21] |
| Severe food insecurity | 2.36*** | 1.68*** | 3.79*** | 2.27*** |
|  | [1.98,2.82] | [1.29,2.20] | [2.50,5.74] | [1.84,2.80] |
| Vuong test of zip vs. standard Poisson: | 6.10 | 8.88 | 6.07 | 10.06 |
| N | 4795 | 4795 | 4795 | 4795 |

**Notes** Incidence rate ratio; 95% confidence intervals in brackets

All models were weighted with post-stratification sampling weights and adjusted for age, sex, residence, ethnicity, province, education, number of children, marital status, number of adults, health insurance participation, family income, fixed assets, liquid assets, debt, smoking status, and drinking frequency.

^*^ *p* < 0.05, ^**^ *p* < 0.01, ^***^ *p* < 0.001

**Supplementary Table M**

The correlation between 4-category household food insecurity and health status (excluding family income and liquid assets)

|  | **(1)** | **(2)** | **(3)** | **(4)** |
| --- | --- | --- | --- | --- |
|  | **Any chronic condition** | **Any mental disorder** | **Any disability** | **Any doctor visit** |
| Food security | 1.00 | 1.00 | 1.00 | 1.00 |
|  | [1.00,1.00] | [1.00,1.00] | [1.00,1.00] | [1.00,1.00] |
| Mild food insecurity | 1.97*** | 2.13*** | 1.90* | 1.62*** |
|  | [1.47,2.63] | [1.46,3.12] | [1.16,3.12] | [1.22,2.15] |
| Moderate food insecurity | 3.62*** | 5.45*** | 5.72*** | 2.18*** |
|  | [2.17,6.07] | [3.24,9.17] | [3.31,9.89] | [1.37,3.48] |
| Severe food insecurity | 1.91 | 6.37*** | 7.28*** | 2.70** |
|  | [0.96,3.82] | [3.43,11.82] | [3.46,15.28] | [1.34,5.44] |
| N | 4795 | 4795 | 4795 | 4795 |

**Notes** Adjusted odds ratios; 95% confidence intervals in brackets

All models were weighted with post-stratification sampling weights and adjusted for age, sex, residence, ethnicity, province, education, number of children, marital status, number of adults, health insurance participation, fixed assets, debt, smoking status, and drinking frequency.

^*^ *p* < 0.05, ^**^ *p* < 0.01, ^***^ *p* < 0.001

**Supplementary Table N**

The correlation between binary food insecurity status and chronic conditions

| **Chronic conditions** | **Food insecurity** |
| --- | --- |
| Hypertension | 1.53*** [1.22,1.91] |
| Hyperlipidemia | 1.57*** [1.21,2.03] |
| Hyperglycemia | 1.52* [1.09,2.12] |
| Diabetes | 1.37 [0.88,2.13] |
| Malnutrition | 2.43*** [1.76,3.36] |
| Anaemia | 2.05*** [1.67,2.51] |
| Gout/Hyperuricemia | 1.37 [0.97,1.95] |
| Osteoporosis | 1.86*** [1.29,2.70] |
| Intervertebral Disc Disease | 1.69*** [1.34,2.12] |
| Tumor/Cancer | 1.53 [0.70,3.33] |
| Cerebrovascular Disease | 1.85 [0.73,4.69] |
| Heart Disease | 1.90* [1.06,3.42] |
| Respiratory Disease | 2.29*** [1.42,3.71] |
| Gastrointestinal Disease | 1.85*** [1.47,2.32] |
| Urinary System Disease | 2.52** [1.34,4.77] |
| Endocrine System Disease | 2.22*** [1.49,3.31] |
| Rheumatic Disease | 1.95** [1.19,3.18] |
| Neurological Disorders | 3.93** [1.62,9.50] |

**Notes** Adjusted odds ratios; 95% confidence intervals in brackets.

All models were weighted with post-stratification sampling weights and adjusted for age, sex, residence, ethnicity, province, education, number of children, marital status, number of adults, health insurance participation, family income, fixed assets, liquid assets, debt, smoking status, and drinking frequency.

^*^ *p*<0.05, ^**^ *p*<0.01, ^***^ *p*<0.001

The relationship between food insecurity and tumour cancer, cerebrovascular disease, urinary system disease, neurological disorder disease are based on the extremely small sample size. The results may be unstable and should be interpreted with caution.

**Supplementary Table O**

The correlation between binary food insecurity status and mental disorders

| **Mental disorders** | **Food insecurity** |
| --- | --- |
| Major Depression | 3.15*** [2.27,4.36] |
| General Anxiety Disorder | 2.62*** [2.04,3.38] |
| Bipolar disorder | 2.57** [1.37,4.83] |
| Insomnia | 2.32*** [1.82,2.95] |
| Obsessive-Compulsive Disorder(ocd) | 2.89*** [1.86,4.47] |
| Schizophrenia | 3.34** [1.63,6.86] |
| Personality Disorder | 3.29* [1.30,8.31] |
| Anorexia | 2.49** [1.31,4.74] |

**Notes** Adjusted odds ratios; 95% confidence intervals in brackets.

All models were weighted with post-stratification sampling weights and adjusted for age, sex, residence, ethnicity, province, education, number of children, marital status, number of adults, health insurance participation, family income, fixed assets, liquid assets, debt, smoking status, and drinking frequency.

^*^ *p*<0.05, ^**^ *p*<0.01, ^***^ *p*<0.001

The relationship between food insecurity and bipolar disorder, schizophrenia, personality disorder, anorexia are based on the extremely small sample size. The results may be unstable and should be interpreted with caution.

**Supplementary Table P**

The correlation between binary food insecurity status and disability

| **Disabilities** | **Visual Disability** | **Speech - hearing Disability** | **Intellectual Disability** | **Physical Disability** | **Mental Disability** |
| --- | --- | --- | --- | --- | --- |
| **Food insecurity** | 2.53*** | 3.35*** | 10.68*** | 2.21*** | 3.31*** |
|  | [1.70,3.77] | [2.00,5.63] | [5.06,22.52] | [1.55,3.16] | [1.90,5.75] |

**Notes** Adjusted odds ratios; 95% confidence intervals in brackets.

All models were weighted with post-stratification sampling weights and adjusted for age, sex, residence, ethnicity, province, education, number of children, marital status, number of adults, health insurance participation, family income, fixed assets, liquid assets, debt, smoking status, and drinking frequency.

^*^ *p*<0.05, ^**^ *p*<0.01, ^***^ *p*<0.001

The relationship between food insecurity and Intellectual disability, Mental disability are based on the extremely small sample size. The results may be unstable and should be interpreted with caution.

**Supplementary Table Q**

The correlation between binary food insecurity status and doctor visits

| **Doctor visits** | **Due to Chronic Conditions** | **Due to Acute Conditions** | **Due to Mental Disorders** | **Due to Unintentional Injuries** | **Due to Intentional Injuries** |
| --- | --- | --- | --- | --- | --- |
| **Food insecurity** | 2.04*** | 1.53*** | 4.66*** | 1.79*** | 1.93* |
|  | [1.56,2.68] | [1.29,1.80] | [3.09,7.02] | [1.45,2.22] | [1.16,3.21] |

**Notes** Adjusted odds ratios; 95% confidence intervals in brackets.

All models were weighted with post-stratification sampling weights and adjusted for age, sex, residence, ethnicity, province, education, number of children, marital status, number of adults, health insurance participation, family income, fixed assets, liquid assets, debt, smoking status, and drinking frequency.

^*^ *p*<0.05, ^**^ *p*<0.01, ^***^ *p*<0.001

**Supplementary Table R**

The interaction effect of binary food insecurity status and health insurance on health (logistic model)

|  | **(1)** | **(2)** | **(3)** | **(4)** |
| --- | --- | --- | --- | --- |
|  | **Any chronic condition** | **Any mental disorder** | **Any disability** | **Any doctor visit** |
| **Main** | | | | |
| Food security | 1.00 | 1.00 | 1.00 | 1.00 |
|  | [1.00,1.00] | [1.00,1.00] | [1.00,1.00] | [1.00,1.00] |
| Food insecurity | 9.04*** | 3.78* | 4.59* | 4.04** |
|  | [3.17,25.82] | [1.14,12.50] | [1.24,17.01] | [1.45,11.24] |
| **Health insurance** | | | | |
| Uninsured | 1.00 | 1.00 | 1.00 | 1.00 |
|  | [1.00,1.00] | [1.00,1.00] | [1.00,1.00] | [1.00,1.00] |
| UEBMI | 3.07* | 1.15 | 0.73 | 2.23 |
|  | [1.30,7.25] | [0.41,3.23] | [0.23,2.33] | [0.95,5.22] |
| URRBMI | 2.82* | 1.88 | 1.41 | 2.33 |
|  | [1.18,6.75] | [0.66,5.36] | [0.44,4.56] | [0.98,5.50] |
| Only other types of health insurance | 2.81* | 2.23 | 1.69 | 2.07 |
|  | [1.09,7.25] | [0.70,7.04] | [0.45,6.36] | [0.80,5.35] |
| **Food insecurity# health insurance** | | | | |
| Food insecurity#  uninsured | 1.00 | 1.00 | 1.00 | 1.00 |
|  | [1.00,1.00] | [1.00,1.00] | [1.00,1.00] | [1.00,1.00] |
| Food insecurity#UEBMI | 0.21** | 0.76 | 0.60 | 0.44 |
|  | [0.07,0.62] | [0.23,2.56] | [0.16,2.30] | [0.16,1.24] |
| Food insecurity#URRBMI | 0.26* | 0.66 | 0.56 | 0.58 |
|  | [0.09,0.78] | [0.19,2.29] | [0.14,2.19] | [0.20,1.67] |
| Food insecurity#only other types of health insurance | 0.21* | 0.58 | 0.47 | 0.55 |
|  | [0.06,0.71] | [0.14,2.38] | [0.09,2.35] | [0.16,1.88] |
| N | 4795 | 4795 | 4795 | 4795 |

**Notes** Adjusted odds ratios; 95% confidence intervals in brackets.

All models were weighted with post-stratification sampling weights and adjusted for age, sex, residence, ethnicity, province, education, number of children, marital status, number of adults, family income, fixed assets, liquid assets, debt, smoking status, and drinking frequency.

^*^ *p*<0.05, ^**^ *p*<0.01, ^***^ *p*<0.001

**Supplementary Table S**

The interaction effect of binary food insecurity status and health insurance on health (Possion model)

|  | **(1)** | **(2)** | **(3)** | **(4)** |
| --- | --- | --- | --- | --- |
|  | **Number of chronic conditions** | **Number of mental disorders** | **Number of disabilities** | **Number of reasons for doctor visits** |
| **Main** | | | | |
| Food security | 1.00 | 1.00 | 1.00 | 1.00 |
|  | [1.00,1.00] | [1.00,1.00] | [1.00,1.00] | [1.00,1.00] |
| Food insecurity | 6.24*** | 4.73** | 6.22** | 3.67** |
|  | [2.38,16.35] | [1.79,12.46] | [1.95,19.87] | [1.69,7.97] |
| **Health insurance** | | | | |
| Uninsured | 1.00 | 1.00 | 1.00 | 1.00 |
|  | [1.00,1.00] | [1.00,1.00] | [1.00,1.00] | [1.00,1.00] |
| UEBMI | 2.21 | 1.31 | 0.95 | 2.17* |
|  | [0.94,5.17] | [0.55,3.14] | [0.32,2.81] | [1.11,4.25] |
| URRBMI | 2.24 | 2.24 | 1.71 | 2.32* |
|  | [0.95,5.28] | [0.92,5.44] | [0.57,5.12] | [1.18,4.58] |
| Only other types of health insurance | 2.35 | 2.33 | 2.16 | 1.91 |
|  | [0.94,5.84] | [0.86,6.33] | [0.61,7.70] | [0.92,3.98] |
| **Food insecurity# health insurance** | | | | |
| Food insecurity#  uninsured | 1.00 | 1.00 | 1.00 | 1.00 |
|  | [1.00,1.00] | [1.00,1.00] | [1.00,1.00] | [1.00,1.00] |
| Food insecurity#UEBMI | 0.26** | 0.55 | 0.50 | 0.43* |
|  | [0.10,0.67] | [0.20,1.50] | [0.15,1.67] | [0.20,0.95] |
| Food insecurity#URRBMI | 0.28* | 0.41 | 0.38 | 0.45* |
|  | [0.10,0.74] | [0.15,1.14] | [0.11,1.30] | [0.20,1.00] |
| Food insecurity#only other types of health insurance | 0.23** | 0.34 | 0.29 | 0.52 |
|  | [0.08,0.65] | [0.11,1.09] | [0.07,1.25] | [0.22,1.27] |
| N | 4795 | 4795 | 4795 | 4795 |

**Notes** Incidence rate ratio; 95% confidence intervals in brackets.

All models were weighted with post-stratification sampling weights and adjusted for age, sex, residence, ethnicity, province, education, number of children, marital status, number of adults, family income, fixed assets, liquid assets, debt, smoking status, and drinking frequency.

^*^ *p*<0.05, ^**^ *p*<0.01, ^***^ *p*<0.001

**Supplementary Table T**

Predicted probability of number of chronic conditions and number of reasons for doctor visits for food-secure and food- insecure individuals

|  | **(1)** | **(2)** | **(3)** |
| --- | --- | --- | --- |
|  | **Any chronic condition** | **Number of chronic conditions** | **Number of reasons for doctor visits** |
| food security | 0.40*** | 0.63*** | 0.35*** |
|  | (0.01) | (0.02) | (0.01) |
| food insecurity | 0.57*** | 1.04*** | 0.58*** |
|  | (0.01) | (0.04) | (0.02) |
|  |  |  |  |
| uninsured | 0.33*** | 0.77*** | 0.30*** |
|  | (0.05) | (0.15) | (0.05) |
| UEBMI | 0.45*** | 0.75*** | 0.42*** |
|  | (0.01) | (0.02) | (0.01) |
| URRBMI | 0.45*** | 0.79*** | 0.45*** |
|  | (0.02) | (0.04) | (0.02) |
| only other types of health insurance | 0.43*** | 0.76*** | 0.40*** |
|  | (0.04) | (0.09) | (0.04) |
|  |  |  |  |
| food secure#uninsured | 0.19** | 0.29* | 0.16** |
|  | (0.06) | (0.12) | (0.06) |
| food secure#UEBMI | 0.41*** | 0.63*** | 0.35*** |
|  | (0.01) | (0.02) | (0.01) |
| food secure#URRBMI | 0.39*** | 0.64*** | 0.38*** |
|  | (0.02) | (0.05) | (0.03) |
| food secure#only other types of health insurance | 0.39*** | 0.67*** | 0.31*** |
|  | (0.05) | (0.12) | (0.05) |
|  |  |  |  |
| food insecure#uninsured | 0.66*** | 1.78*** | 0.60*** |
|  | (0.07) | (0.41) | (0.13) |
| food insecure#UEBMI | 0.56*** | 1.01*** | 0.56*** |
|  | (0.02) | (0.05) | (0.02) |
| food insecure#URRBMI | 0.59*** | 1.12*** | 0.62*** |
|  | (0.03) | (0.08) | (0.04) |
| food insecure#only other types of health insurance | 0.53*** | 0.96*** | 0.60*** |
|  | (0.06) | (0.15) | (0.09) |
| N | 4795 | 4795 | 4795 |

**Notes** Standard errors in parentheses.

All models were weighted with post-stratification sampling weights and adjusted for age, sex, residence, ethnicity, province, education, number of children, marital status, number of adults, family income, fixed assets, liquid assets, debt, smoking status, and drinking frequency.

^*^ *p* < 0.05, ^**^ *p* < 0.01, ^***^ *p* < 0.001

**Supplementary Table U**

The mediating effect of key pathways on the food insecurity-health relationship

|  | **Odds**  **ratio** | **Indirect effects**  **(odds ratio)** | | |
| --- | --- | --- | --- | --- |
| **Mediator** |  | **Mild food**  **insecurity** | **Moderate food insecurity** | **Severe food**  **insecurity** |
| **Outcome = Any chronic condition** | | | | |
| Health care delayed or forgone due to cost | 2.06* | 4.09* | 14.80* | 18.13* |
| Diminished food expenditures | 0.78 | 0.85 | 1.14 | 0.57 |
| Subjective cognition of dietary health | 0.47* | 0.17* | 0.17* | 0.08* |
| Social support deficit | 1.61*** | 3.80*** | 6.20*** | 9.53*** |
| Psychological chronic stress | 3.10*** | 6.03*** | 14.54*** | 6.49*** |
| **Outcome = Any mental disorder** | | | | |
| Health care delayed or forgone due to cost | 2.12** | 4.25** | 15.30** | 18.74** |
| Diminished food expenditures | 0.68 | 0.75 | 1.00 | 0.50 |
| Subjective cognition of dietary health | 0.64* | 0.33* | 0.20* | 0.17* |
| Social support deficit | 3.12*** | 7.38*** | 12.03*** | 18.51*** |
| Psychological chronic stress | 6.10*** | 11.85*** | 28.60*** | 12.75*** |
| **Outcome = Any disability** | | | | |
| Health care delayed or forgone due to cost | 2.27* | 5.64* | 17.51* | 21.19* |
| Diminished food expenditures | 0.81 | 0.88 | 1.18 | 0.59 |
| Subjective cognition of dietary health | 1.02 | 0.53 | 0.31 | 0.28 |
| Social support deficit | 2.35** | 5.55** | 9.04** | 13.91** |
| Psychological chronic stress | 2.62*** | 5.09*** | 12.27*** | 5.48*** |
| **Outcome = Any doctor visit** | | | | |
| Health care delayed or forgone due to cost | 2.06** | 5.12** | 15.90** | 18.25** |
| Diminished food expenditures | 0.71 | 0.77 | 1.03 | 0.51 |
| Subjective cognition of dietary health | 0.67** | 0.34** | 0.20** | 0.18** |
| Social support deficit | 1.23 | 2.91 | 3.74 | 6.28 |
| Psychological chronic stress | 2.45*** | 4.77*** | 11.49*** | 5.13*** |
| N | 4795 | 976 | 293 | 127 |

**Notes** Adjusted odds ratios; 95% confidence intervals in brackets.

All models were weighted with post-stratification sampling weights and adjusted for age, sex, residence, ethnicity, province, education, number of children, marital status, number of adults, health insurance participation, family income, fixed assets, liquid assets, debt, smoking status, and drinking frequency.

^*^ *p*<0.05, ^**^ *p*<0.01, ^***^ *p*<0.001

**Supplementary Table V**

**The GSEM models' AIC and BIC differences**

|  | **AIC** | **BIC** |
| --- | --- | --- |
| Any chronic condition | 8051.16 | 8653.36 |
| Any mental disorder | 5221.26 | 5823.47 |
| Any disability | 4455.62 | 5057.83 |
| Any doctor visit | 7746.56 | 8348.77 |
